# Angiogenic and inflammatory responses in human induced microglia-like (iMG) cells from patients with Moyamoya disease

**DOI:** 10.1038/s41598-023-41456-z

**Published:** 2023-09-08

**Authors:** Noritoshi Shirozu, Masahiro Ohgidani, Nobuhiro Hata, Shunya Tanaka, Shogo Inamine, Noriaki Sagata, Tetsuaki Kimura, Ituro Inoue, Koichi Arimura, Akira Nakamizo, Ataru Nishimura, Naoki Maehara, Soh Takagishi, Katsuma Iwaki, Tomohiro Nakao, Keiji Masuda, Yasunari Sakai, Masahiro Mizoguchi, Koji Yoshimoto, Takahiro A. Kato

**Affiliations:** 1 Department of Neurosurgery, Graduate School of Medical Sciences, Kyushu University, Fukuoka, Japan; 2 Department of Neuropsychiatry, Graduate School of Medical Sciences, Kyushu University, Maidashi 3-1-1, Higashi-ku, Fukuoka, 812-8582 Japan; 3 Department of Functional Anatomy and Neuroscience, Asahikawa Medical University, Asahikawa, Japan; 4 Division of Human Genetics, National Institute of Genetics, Mishima, Japan; 5 Medical Genome Center, Research Institute, National Center for Geriatrics and Gerontology, Obu, Japan; 6 Section of Oral Medicine for Children, Division of Oral Health, Growth and Development, Faculty of Dental Science, Kyushu University, Fukuoka, Japan; 7 Department of Pediatrics, Graduate School of Medical Sciences, Kyushu University, Fukuoka, Japan

**Keywords:** Microglia, Neuro-vascular interactions

## Abstract

Angiogenic factors associated with Moyamoya disease (MMD) are overexpressed in M2 polarized microglia in ischemic stroke, suggesting that microglia may be involved in the pathophysiology of MMD; however, existing approaches are not applicable to explore this hypothesis. Herein we applied blood induced microglial-like (iMG) cells. We recruited 25 adult patients with MMD and 24 healthy volunteers. Patients with MMD were subdivided into progressive (N = 7) or stable (N = 18) group whether novel symptoms or radiographic advancement of Suzuki stage within 1 year was observed or not. We produced 3 types of iMG cells; resting, M1-, and M2-induced cells from monocytes, then RNA sequencing followed by GO and KEGG pathway enrichment analysis and qPCR assay were performed. RNA sequencing of M2-induced iMG cells revealed that 600 genes were significantly upregulated (338) or downregulated (262) in patients with MMD. Inflammation and immune-related factors and angiogenesis-related factors were specifically associated with MMD in GO analysis. qPCR for *MMP9*, *VEGFA*, and *TGFB1* expression validated these findings. This study is the first to demonstrate that M2 microglia may be involved in the angiogenic process of MMD. The iMG technique provides a promising approach to explore the bioactivity of microglia in cerebrovascular diseases.

## Introduction

Moyamoya disease (MMD) is a chronic disease of unknown cause in which the terminal portion of the internal carotid artery is progressively narrowed and occluded, and an abnormal vascular network develops at the base of the brain^1–3^. Recently, *RNF213* was identified as a susceptibility gene for MMD. However, the mechanism by which *RNF213* mutation is involved in the pathogenesis of MMD is yet to be unveiled^4,5^.

Various biomarkers and angiogenic factors, including cytokines such as vascular endothelial growth factor (VEGFA) and matrix metalloproteinase (MMP9), are involved in MMD^6,7^. In addition, microglia are immune cells in the brain that are an important source of cytokines and are implicated in a variety of brain diseases^8,9^. The activation status of microglia can be generally divided into M1 and M2 and activated M2 microglia play an anti-inflammatory role by phagocytosing cellular debris and damaged neuron^10,11^. Interestingly, VEGF and MMP9 are known to be highly expressed in M2-polarized microglia, which promotes angiogenesis and helps repair damage caused by ischemic stroke^12,13^. M2 responses/markers have been implicated in MMD pathogenesis before^14–16^.

Based on these data, we hypothesize that microglia are involved in MMD and ischemic stroke. In vivo assays for analyzing human microglia bioactivity are not feasible owing to ethical complications. As an alternative approach, an *RNF213*-deficient mouse model would be expected to be a suitable tool for testing this hypothesis. However, previous studies reported that this model did not reproduce the phenotype of MMD intracranially, and internal carotid artery ligation in knock-in mice with the mutation did not show any specific phenotype compared with that in the wild-type mice^17,18^. Therefore, it is difficult to test the hypothesis using an animal model of MMD, and to date, there are no investigations to show the relationship between microglia and MMD.

Recently, We have originally developed a technique to create directly induced microglia-like (iMG) cells from fresh human peripheral blood monocytes adding GM-CSF and IL-34 for 2 weeks, instead of PET, brain biopsy and iPS technique^19–21^. Clinical conditions of numerous neuropsychiatric disorders can be predicted by evaluating microglial activity using iMG cells as surrogates^19–24^. RNA-seq analysis revealed that primary microglia (pMG) cultured from human brain tissue harvested during surgery from patients with a neurological disorder showed similar properties to iMG cells derived from the same patient at the same time^25^. Furthermore, analysis of microglial marker expression by RNA-seq and other methods showed disease-specific behavior^25^. These results indicate that patients-derived iMG cells will take a role as one of the important surrogate markers to predict brain microglial conditions in human.

Therefore, we hypothesized that we could explore the molecular and cellular mechanisms underlying the role of microglia in patients with MMD through the analysis of *RNF213* in iMG cells to generate new prognostic predictions and therapeutic strategies. In this study, we investigated gene expression profiles in iMG cells derived from patients with MMD as the first exploratory case–control study.

## Materials and methods

### Study subjects

All experiments performed were approved by the Ethics Committee of the Graduate School of Medicine of Kyushu University, which was conducted in accordance with the ethical principles of the Belmont Report (application number: 895-00 and 29-624). All studies were conducted in accordance with relevant guidelines/regulations and informed consent was obtained from all participants in accordance with the Declaration of Helsinki.

The following were included in the study: (1) patients diagnosed with MMD by digital subtraction angiography (DSA), (2) patients who were at least 20 years old at the time consent was obtained, and (3) patients without a cerebral infarction or hemorrhage within 3 months and had not undergone DSA within 3 months. For this study, only those aged 20 and older were targeted.

Based on the cohort study design, from September 2019 to April 2022, upon among the 19,136 people who visited the Department of Neurosurgery of Kyushu University Hospital, 87 adult patients with MMD were identified. Among them, 35 were available for recruitment and asked for informed consent to participate in the study, and 25 gave their consent. Note that none withdrew from the study.

To stratify MMD by disease status, we classified patients who showed progression of disease stage according to Suzuki stages by imaging tests (MRI, DSA) or symptoms (transient ischemic attack [TIA], cerebral infarction) within 1 year into the progressive group, and patients who showed no progression of Suzuki stage for more than 1 year and more than 1 year since their last attack were classified into the stable group.

In addition, 24 healthy persons recruited at the Kyushu University Hospital campus and provided informed consent were included as the control group.

### Induction of microglia-like (iMG) cells from human peripheral blood

Peripheral blood was collected from patients with MMD and healthy participants into heparin tubes. Peripheral blood mononuclear cells (PBMCs) were separated by density gradient centrifugation in Histopaque-1077 (Sigma Chemical Co., St. Louis, MO, USA); PBMCs were then transferred to RPMI-1640 (Nacalai Tesque, Kyoto, Japan) medium containing 10% heat-inactivated fetal bovine serum. PBMCs were plated in culture chambers at a density of 4 × 10^5^ cells/mL and incubated overnight under standard culture conditions (37 °C, 5% CO_2_). After overnight incubation, the culture supernatant and non-adherent cells were removed. Adherent cells (monocytes) were cultured in RPMI-1640 Glutamax (Invitrogen) supplemented with 1% antibiotic/antifungal, recombinant human GM-CSF (10 ng/mL; R&D Systems, Minneapolis, USA) and recombinant human IL-34 (100 ng/mL; R&D Systems) for 14 days to generate iMG cells. Then, resting with nothing added, M1 microglia were induced by adding IFNG (100 ng/mL; R&D Systems), whereas M2 were induced with IL-4 (10 ng/mL; R&D Systems), and the two were cultured for another day^19,20^. Human iMG cells have been validated to be similar to human microglia, using RNA sequencing and cell surface markers^19–24^.

### Direct sequencing analysis of RNF213

*RNF213* encodes a 5,256 amino acid protein with AAA-ATPase and RING-finger E3 ubiquitin ligase domains^26^. Recently, the c.14576G > A polymorphism in *RNF213* located in the 17q25-ter region was reported to be found in 95% of patients with familial MMD and 79% of patients with non-familial MMD, identifying *RNF213* as a susceptibility gene for MMD^27,28^. Genomic DNA was extracted from blood samples. DNA was amplified using a GeneAmp 9700 thermal cycler (Applied Biosystems, Foster City, CA, USA) with forward primer 5′-CTGCATCACAGGAAATGACACTG, reverse primer 5′-TGACGAGAAGAGCTTTCAGACGA, which produced a 783 bp fragment^29,30^. Genotyping of *RNF213* p.R4810K was analyzed by an automated sequencer equipped with a 3730 DNA analyzer (Applied Biosystems, Foster City, CA, USA). Analysis of results was performed using MEGAx software version 10.2.4.

### RNA sequencing

First, to compare differences in gene expression patterns in resting iMG cells derived from patients with MMD (N = 22) and healthy controls (N = 20), RNA sequencing was performed.

Next, to compare differences in gene expression patterns in M2-induced iMG cells between patients with MMD (progressive group, stable group) and healthy controls, RNA sequencing was performed using 4 samples from each group to match the conditions (age and sex) because the number of cases was small (four cases).

The sequencing libraries were prepared from 1 μg of total RNA with NEBNext Ultra Directional RNA Library Prep Kit for Illumina according to the manufacturer’s instructions. Cluster amplification and 150 bp paired-end sequencing were performed according to the manufacturer’s protocol (Illumina) for NovaSeq (Illumina, San Diego, California, USA).

### RNA-seq data analysis

All read data were checked with FastQC (v0.11.7) and trimmed using Trimmomatic (v-0.38). The sequencing results were analyzed using RNA-seq (https://ranaseq.eu.), which quantifies FASTQ files, reads trimming, quality control of samples, differential expression tests, functional enrichment analysis, and gene set expression analysis^31^. The significantly differentially expressed genes (DEGs) (log2FC > 0, *p*-value < 0.05) were visualized heatmaps and volcano plot. For the gene set enrichment analysis (GSEA), the fgsea R package was integrated. Then gene expression and GSEA between patients with MMD and healthy controls and between the progressive and stable groups of MMD were compared using RaNA-seq. The significant enrichment threshold was set as *p*-value < 0.05.

### Phagocytosis experiments on iMG cells

Fluorescent labeled latex beads (Latex Beads-FITC-Rabbit IgG-complex, Cayman Chemical) were added to iMG cells generated from 14-day cultures and cultured overnight. Cells were then collected by Cellotion (Takara Bio), and the fluorescence intensity of the cell suspension was measured using a Tali Image-Based Cytometer (Invitrogen).

### Quantitative reverse transcription-polymerase chain reaction (qRT-PCR)

To evaluate gene expression patterns in iMG cells, qRT-PCR was performed using a CFX Connect Real-Time PCR Detection System (BioRad, Hercules, CA, USA). iMG cells were washed, total RNA was extracted using the High Pure RNA Isolation kit and reverse-transcribed into cDNA using ReverTra Ace qPCR RT Kit (TOYOBO). qRT-PCR was performed based on primers from predesigned qPCR assays. Normalization was performed using the reference genes glyceraldehyde-3-phosphate dehydrogenase (GAPDH) and beta-2-microglobulin (B2M) from Housekeeping Reference Gene Assays (Integrated DNA Technologies), and the ΔΔ Ct method was used for this study (Table S1).

### Statistical analysis

Results are expressed as mean ± standard deviation. All statistical analyses were performed using JMP software version 16 (Cary, NC: SAS Institute Inc.). The Chi-square or Fisher’s exact test was used to compare the qPCR data. iMG cell correlations were analyzed using Spearman’s correlation coefficient. Statistical significance was determined at the level of α = 0.05. Differences were considered statistically significant when *p* < 0.05.

## Results

### Baseline characteristics

The patient characteristics are shown in Table 1. There was no significant difference in age and sex between the MMD and control groups, but body mass index (BMI) was significantly higher in the MMD group. As for *RNF213* (p.R4810K), there were 6 wild-type, 19 heterozygous, and 0 homozygous cases of MMD, and the carrier rate was 76%. Although previous studies report that the p.R4810K mutation in *RNF213* is carried by about 2.4% of the population in East and Southeast Asia^5,28^, any p.R4810K mutation in *RNF213* was not detected in the analyzed volunteers. Patients with MMD are highly prevalent, especially in Japan (74%^5,32^) and Korea (76%^5,32,33^), which was consistent with our results.

**Table 1:** Clinical features of patients with MMD and control group.

Clinical features	MMD (N = 25)	Control group (N = 24)	*p* value
Mean age (years)	46.8 ± 10.46	41.9 ± 7.81	0.074
Female sex	24 (96%)	19 (79.2%)	0.072
BMI	24.56 ± 0.81	22.04 ± 0.82	**0.034**
Hypertension	9 (18%)	0 (0%)	**0.0011**
Diabetes mellitus	1 (2%)	0 (0%)	0.322
Hyperlipidemia	6 (12%)	0 (0%)	**0.0104**
Smoking	1 (2%)	0 (0%)	0.322
Family history of MMD	2 (8%)		
Supplemental tablet			
Clopidogrel	3 (12%)	0 (0%)	**0.0398**
Cilostazol	4 (16%)	0 (0%)	**0.0167**
Aspirin	12 (48%)	0 (0%)	** < 0.0001**
Levetiracetam	1 (2%)	0 (0%)	0.322
Phenytoin phenobarbital	1 (2%)	0 (0%)	0.322
*RNF213* Genotype*	6/19/0	24/0/0	
Carrier rate (%)	76%	0%	
Suzuki stage			
I	0		
II	0		
III	6		
IV	17		
V			
VI	1		
1			

*RNF213 p.R4810K wild type/heterozygous/homozygous.

Significant values are in [bold].

The patient backgrounds of the progressive and stable groups are shown in Table 2, with 7 patients in the progressive group and 18 patients in the stable group. There were no significant differences between the groups with respect to age, sex, BMI, and *RNF213* variant (p.R4810K).

**Table 2:** Clinical features of progressive groups and stable groups in patients with MMD.

Clinical Features	MMD (N = 25)		*p* value
	Progressive group (N = 7)	Stable group (N = 18)	
Mean age (years)	48 ± 4.53	46.4 ± 11.97	0.743
Female sex	7 (100%)	17 (94.4%)	0.525
BMI	26.0	24.0	0.342
Hypertension	4 (57.1%)	5 (27.8%)	0.169
Diabetes mellitus	0 (0%)	1 (5.6%)	0.415
Hyperlipidemia	3 (42.9%)	3 (16.7%)	0.169
Smoking	0 (0%)	1 (5.6%)	0.415
Family history of MMD	1 (14.2%)	1 (5.6%)	0.646
Period since last symptoms (month)	3.57	271.5	0.2124
Supplemental tablet			
Clopidogrel	1 (14.3%)	2 (11.1%)	0.829
Cilostazol	2 (28.5%)	2 (11.1%)	0.306
Aspirin	5 (71.4%)	7 (38.9%)	0.139
Levetiracetam	0 (0%)	1 (5.6%)	0.415
Phenytoin phenobarbital	0 (0%)	1 (5.6%)	0.415
*RNF213* Genotype*	3/4/0	3/15/0	0.169
Carrier rate (%)	57.1%	83.3%	

*RNF213 p.R4810K wild type/heterozygous/homozygous.

### RNA sequencing using resting and M2-induced iMG cells

**Figure 1:**
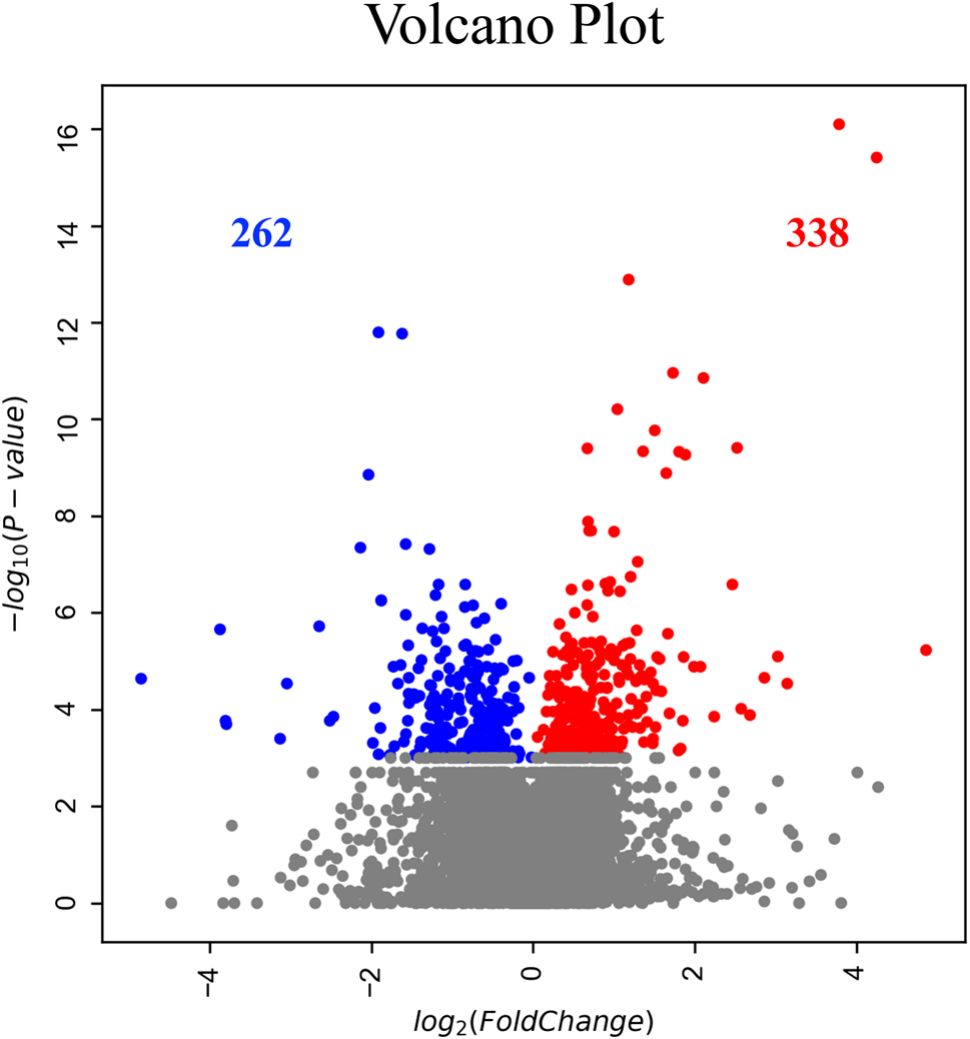
RNA sequencing data in Moyamoya disease (N = 8) and the control group (N = 4) using M2-induced iMG cells. A volcano plot of differential expression results between Moyamoya disease and the control group. It shows the log2 scaled fold change (x-axis) and the − log10 *p*-value (y-axis) of each gene in the differential expression analysis. Genes with significant expression changes in MMD are highlighted as red dots, and the control group is highlighted as blue dots.

**Figure 2:**
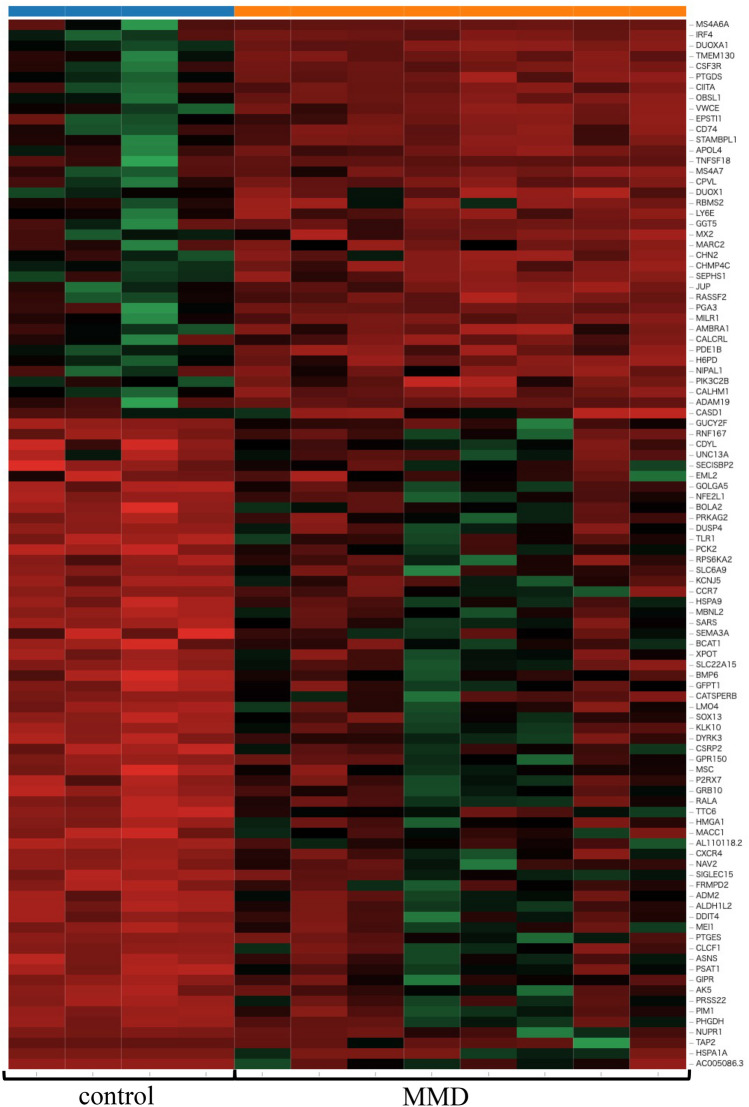
Heatmap of expression values (normalized as transcripts per million) of selected genes between MMD and the control group (showing only the first 100 genes).

**Figure 3:**
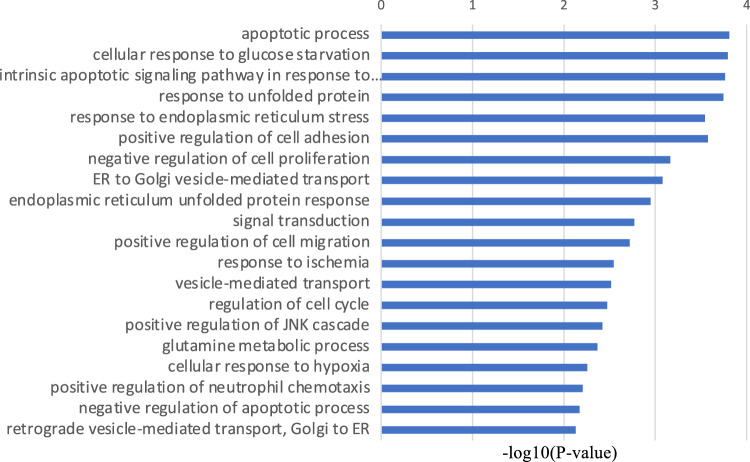
Gene Ontology (GO) terms of biological processes differing between MMD and the control group. It shows the − log10 *p*-value (x-axis) of each GO term of biological processes. The top 20 biological process GO terms that were statistically significantly upregulated in MMD are shown.

First, RNA sequencing was performed using resting iMG cells (from 22 patients with MMD and 20 healthy controls). However, no significant gene expression was found between the control and MMD. Since we hypothesize that M2 microglia may be involved in MMD, we performed RNA sequencing again using M2-induced iMG cells. RNA sequencing data of 24,113 genes were analyzed from M2-induced iMG cells isolated from patients with MMD (N = 8 [including 4 progressive and 4 stable patients]) and compared with those of the control group (N = 4). As a result, we identified 600 significant genes that characterized the patients with MMD, of which 338 and 262 genes were significantly upregulated or downregulated in MMD, respectively (Fig. 1, 2, and Table S2). To further understand the biological processes and pathways, we performed Gene Ontology (GO) and Kyoto Encyclopedia of Genes and Genomes (KEGG) pathway enrichment analysis. According to the functional enrichment results, 69 biological process terms were significantly upregulated, and 35 biological process terms were downregulated in MMD (Table S3 and S4). Factors involved in inflammation and immune-related processes, apoptosis, response to endoplasmic reticulum stress, response to unfolded protein, and positive regulation of neutrophil chemotaxis; ischemia-related factors involved in response to ischemia and the cellular response to hypoxia; and angiogenesis-related factors (positive regulation of angiogenesis) were significantly upregulated in MMD (Fig. 3).

**Figure 4:**
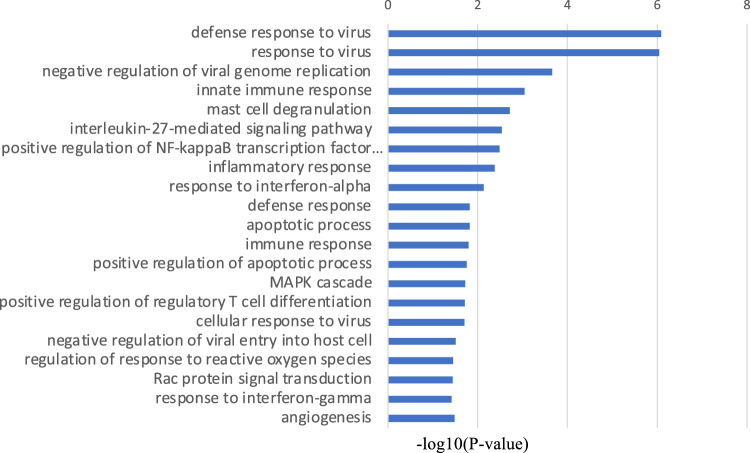
The top 20 biological process GO terms that were statistically significantly downregulated in MMD.

**Figure 5:**
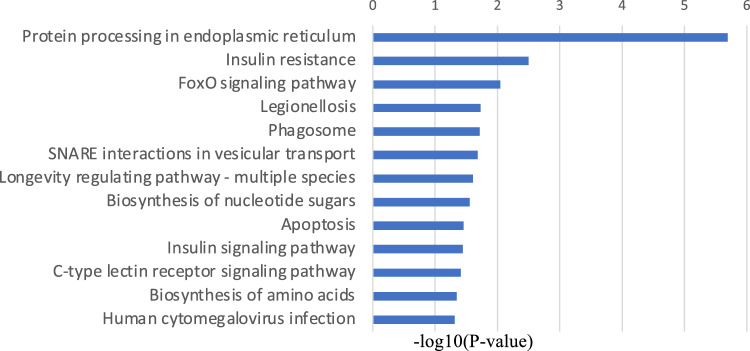
KEGG pathway enrichment analysis of upregulated genes in MMD.

Conversely, the significantly downregulated GO terms in MMD also included inflammation and immune-related factors, such as those mediating the defense response to viruses, immune response, positive regulation of regulatory T cell differentiation, and the positive regulation of NF-kappa B transcription factor activity (Fig. 4). Angiogenesis and inflammatory responses, which are reported to be associated with MMD, were confirmed in both upregulated and downregulated biological process terms in MMD. The KEGG pathway enrichment analysis uncovered that phagosome- and apoptosis-related genes were upregulated in MMD (Fig. 5).

**Figure 6:**
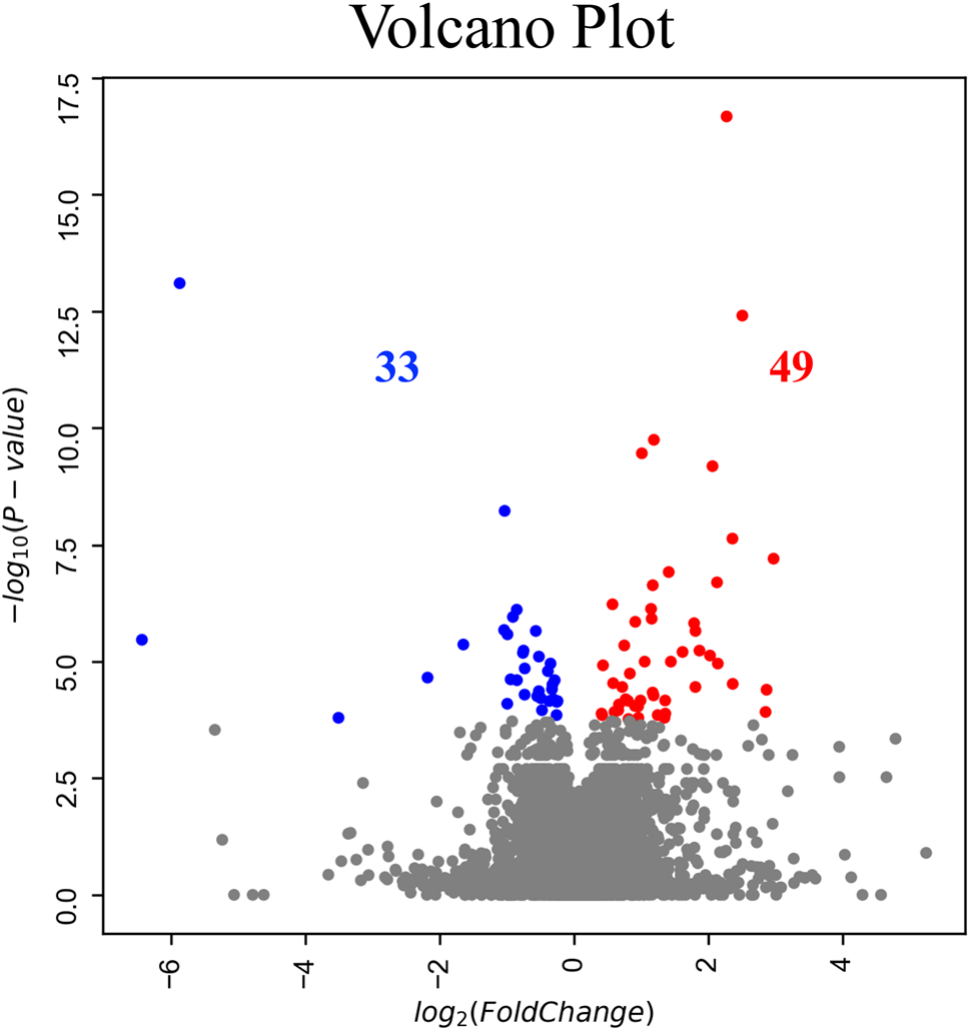
RNA sequencing data in the progressive group (N = 4) and stable group (N = 4) using M2-induced iMG cells. A volcano plot of differential expression results between progressive and stable groups. It shows the log2 scaled fold change (x-axis) and the − log10 *p*-value (y-axis) of each gene in the differential expression analysis. Genes with a significant expression change in the progressive group are highlighted as red dots, and the stable group is highlighted as blue dots.

**Figure 7:**
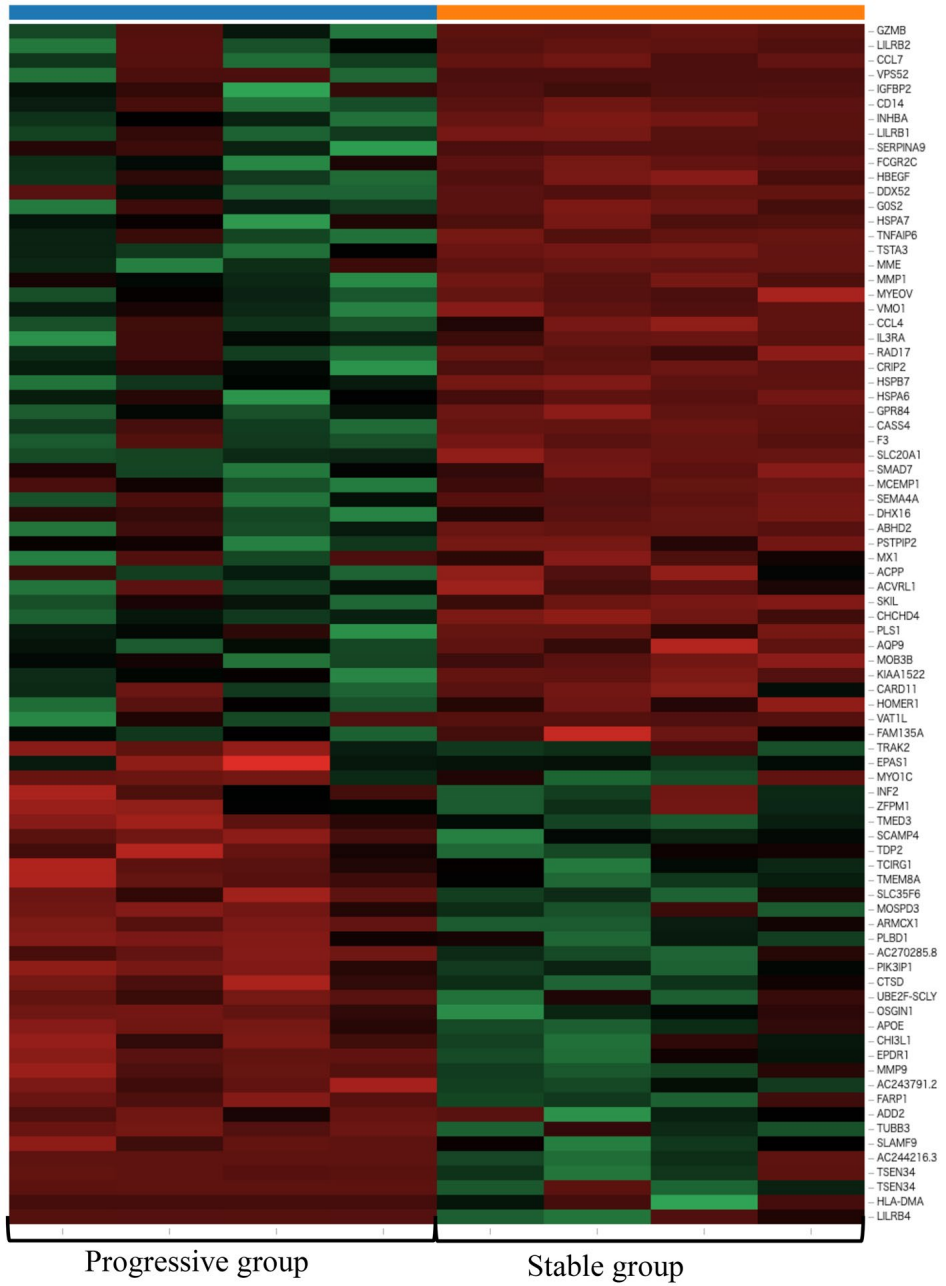
Heatmap of expression values (normalized as transcripts per million) of selected genes between the progressive and stable groups.

**Figure 8:**
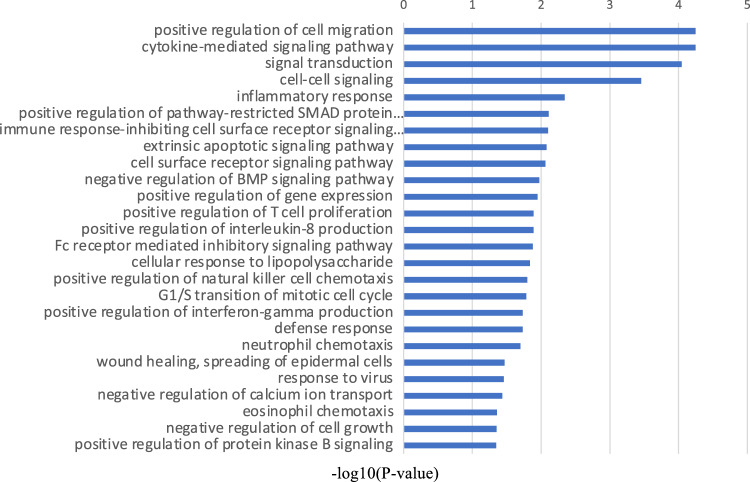
Gene Ontology (GO) is a term for biological processes that differ between the progressive and stable groups in MMD. The graph shows the − log10 *p*-value (x-axis) of each GO term of biological processes. The top 20 biological process GO terms that are statistically significantly upregulated in the progressive group are shown.

**Figure 9:**
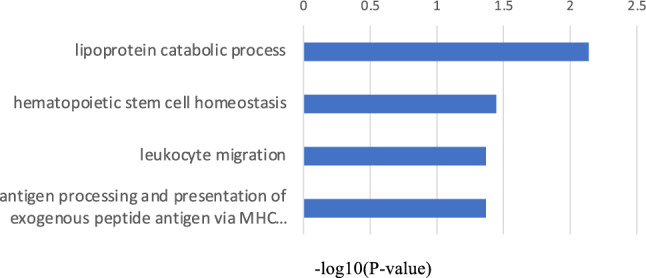
The statistically significantly downregulated GO biological process terms in the progressive group.

**Figure 10:**
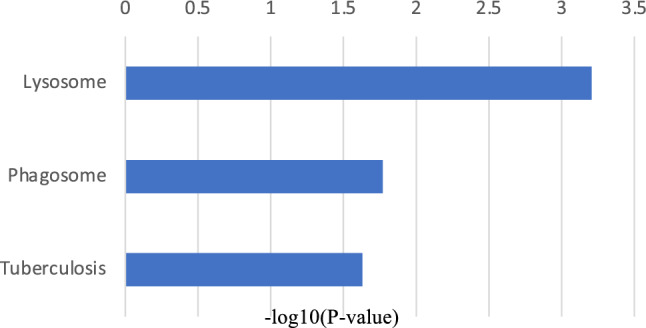
KEGG pathway enrichment analysis of downregulated genes in the progressive group.

Next, RNA sequencing data of 23,088 genes from M2-induced iMG cells were analyzed to compare the progressive group (N = 4) with the stable group (N = 4). As a result, we identified 82 significantly discrepant genes between the two groups, of which 49 and 33 genes were significantly upregulated or downregulated in the progressive group, respectively (Fig. 6, 7 and Table S5). We performed GO and KEGG pathway enrichment analysis to further understand the biological processes and pathways. According to the functional enrichment results, 29 biological process terms were statistically significantly upregulated and 4 biological process terms were downregulated in MMD (Table S6 and S7). Inflammation and immune-related factors from pathways mediating cytokine signaling, positive regulation of natural killer cell chemotaxis, and the defense response were significantly upregulated in the progressive group (Fig. 8). Leukocyte migration was significantly downregulated in the progressive group (Fig. 9). The KEGG pathway enrichment analysis uncovered that phagosome genes were overrepresented among the downregulated genes in the progressive group (Fig. 10).

### RNA expression related to inflammation and angiogenesis (qPCR)

**Figure 11:**
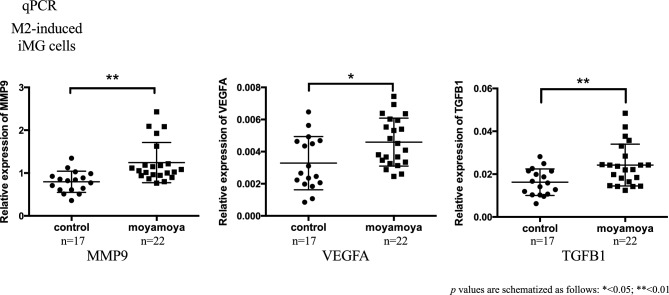
Expression of the angiogenic factors *MMP9*, *VEGFA*, and *TGFB1* was measured by qPCR in M2-induced iMG cells. Fold changes were measured using the ΔΔ Ct method. The relative expression (fold change) of *MMP9*, *TGFB1*, and *VEGFA* was significantly upregulated in M2-induced iMG cells in MMD. (p values are schematized as follows: * < 0.05; ** < 0.01).

The factors most likely to be involved in this study included those related to inflammation and immunity. In addition, several factors involved in angiogenesis were also included. Therefore, we assumed that M2 microglia are deeply involved in inflammation and angiogenesis. To find further validity, the number of samples was increased and RNA expression of inflammation- and angiogenesis-related genes was analyzed in more detail using qPCR. We isolated 3 iMG subtypes (resting, M1-, and M2-induced iMG cells) from each sample and performed qPCR assays. The relative expression level of *RNF213* in these 3 types of cells and monocytes showed no significant difference between MMD and control groups (Fig. S1). However, the upregulation of the angiogenesis factors *MMP9*, *TGFB1*, and *VEGFA* was observed specifically in M2-induced iMG cells derived from patients with MMD (Fig. 11 and S2).

**Figure 12:**
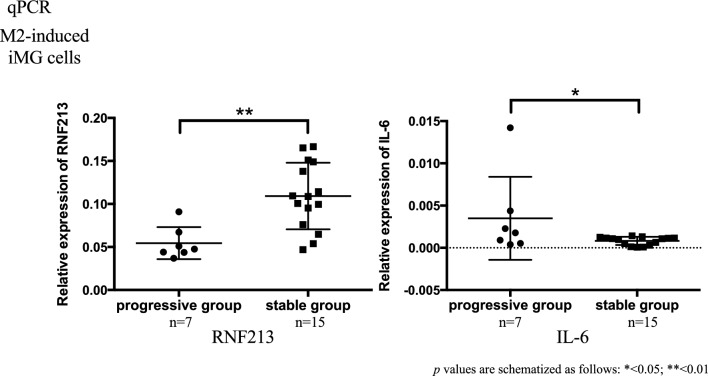
RNA of M2-induced iMG cells was measured by qPCR in the progressive and stable groups. Fold changes were measured using the ΔΔ Ct method. The relative expression (fold change) of *RNF213* expression was significantly lower in the progressive group of M2-induced iMG cells. The relative expression (fold change) of IL-6, an inflammatory cytokine, was significantly upregulated in the progressive group of M2-induced iMG cells.

**Figure 13:**
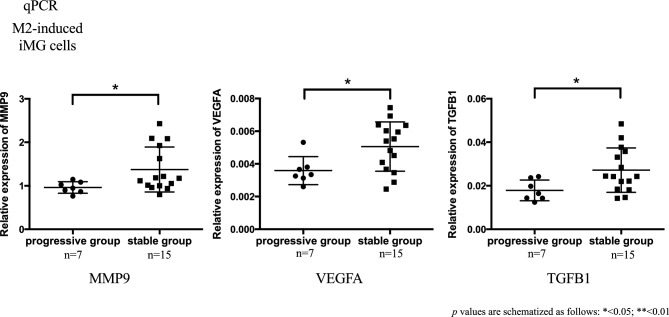
The relative expression (fold change) of *MMP9*, *TGFB1*, and *VEGFA* was significantly upregulated in M2-induced iMG cells in the progressive group. (p values are schematized as follows: * < 0.05; ** < 0.01).

By qPCR, the relative expression of *RNF213* was significantly lower in M2-induced iMG cells of the progressive group. The relative expression of IL-6, an inflammatory cytokine, was significantly upregulated in the progressive group of M2-induced iMG cells (Fig. 12). In M2-induced iMG cells, the relative expression of angiogenic factors such as *MMP9*, *TGFB1*, and *VEGFA* was significantly lower in the progressive group (Figs. 13 and S3). Since the Suzuki stage is also a standard of progressive group, we have compared the *VEGFA* level between different Suzuki stage, but no significant differences were found, with the majority of cases being Suzuki stage 3 and 4.

### Phagocytosis experiments with iMG cells

**Figure 14:**
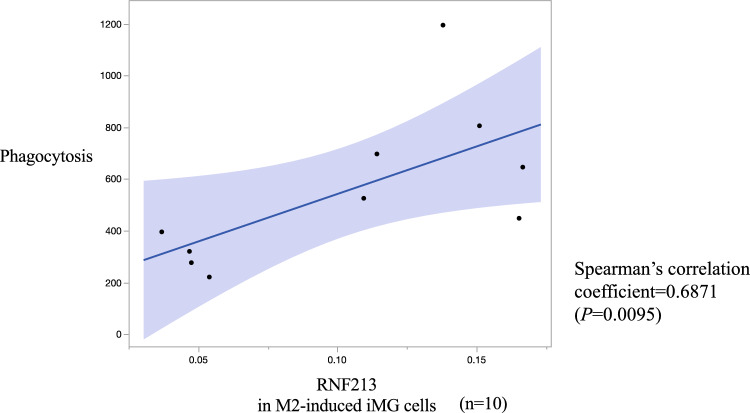
The relative expression (fold change) of *RNF213* in M2-induced iMG cells showed a significant positive correlation with phagocytosis of iMG cells. (Spearman’s correlation coefficient = 0.6871, *p* = 0.0095).

RNA sequencing of genes identified in the KEGG pathway analysis using M2-induced iMG cells showed that phagosome genes were upregulated in MMD, whereas in the progressive group, phagosome genes were downregulated. Based on these results, we performed phagocytosis experiments using surplus iMG cells. The relative expression of *RNF213* in M2-induced iMG cells showed a significant positive correlation with their phagocytic capacity (Fig. 14).

## Discussion

Our GO analysis of RNA sequencing showed that angiogenesis was significantly involved in M2-induced iMG cells of patients with MMD. In addition, qPCR revealed that the relative expression of angiogenic factors (*MMP9*, *VEGFA*, *TGFB1*) was significantly upregulated in these cells compared to the healthy subject group. Interestingly, resting- and M1-microglia showed no significant gene expression differences between patients with MMD and healthy volunteers, which strongly suggests that M2-microglia contribute to the underlying pathophysiology of MMD. This is the first investigation to explore the relationship between the bioactivity of microglia and the clinical state of MMD using patient-derived iMG cells.

Two impaired vessel growth processes are known to be related to MMD: first, proliferative endothelial lesions cause stenosis/occlusion in major cerebral arteries (e.g., internal carotid artery, anterior and middle cerebral artery); second, the formation of fragile perforating arteries, so-called Moyamoya vessels, is thought to compensate for cerebral ischemia and hypoxia^34,35^. Previously, the expression of growth factors such as VEGF, MMP9, TGFB1, and hepatocyte growth factor was found to be higher in serum samples obtained from patients with MMD than those from healthy controls^36^. Overexpression of MMP9 leads to lower vascular statin levels, degradation of vascular basement membranes, and remodeling of the extracellular matrix^37^. This remodeling increases the permeability of the blood–brain barrier, causing intimal proliferation, cerebral ischemia, increased angiogenesis, collateral vessel formation (Moyamoya vessels), and formation and destruction of intracranial aneurysms^37,38^. Elevated plasma VEGFA levels may contribute to collateral vessel formation in MMD^36^. Overexpression of TGFB1 correlates with increased production of extracellular matrix components and accumulation of elastin synthase in smooth muscle cells (SMCs)^36^. MMP9, VEGFA, and TGFB1 are highly expressed in M2-polarized microglia, which can promote angiogenesis and repair damage caused by ischemic stroke^12,13^. M2 responses/markers have been implicated in MMD pathogenesis before^14–16^. Thus, M2 microglia are suggested to be involved in the angiogenesis of Moyamoya vessels that form as compensation for cerebral ischemia and hypoxia. Our results provide a novel insight that supports such hypotheses. The iMG technique should be utilized for further analyses regarding the relationship between microglia and MMD.

To explore our hypothesis that microglia play a certain role in the pathophysiological progression of MMD, the patients with MMD were subdivided into two groups, progressive and stable. The classification was based on Suzuki stages according to characteristics indicating the progression of disease uncovered on imaging tests (MRI, DSA) or the presence of novel symptoms (TIA, cerebral infarction) within 1 year. Thereby, the discrepancies between these two groups were surveyed. In qPCR, the relative expression of *MMP9*, *VEGFA*, and *TGFB1* was significantly lower in M2-induced iMGs in the progressive group compared to the stable group. These results suggest that M2 microglia can downregulate the expression of *MMP9*, *VEGF*, and *TGFB1* and suppress angiogenesis in Moyamoya vessels, inducing the collapse of cerebral hemodynamics resulting in the progression of the MMD state based on our stratification.

**Figure 15:**
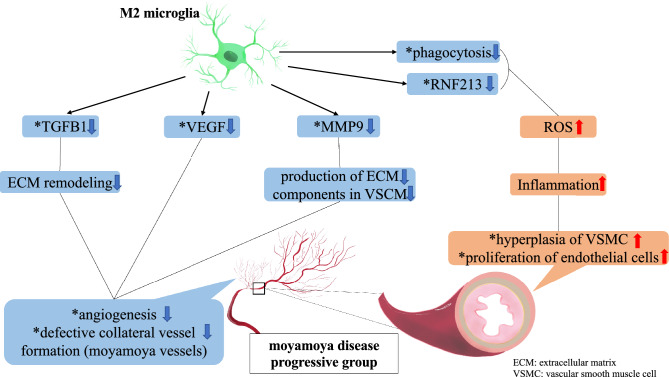
Correlation between M2 microglia and angiogenesis in MMD and hyperplasia of vascular smooth muscle cell in the progressive group. (*: our findings on M2 microglia in this study).

In this study, KEGG pathway enrichment analysis of RNA sequencing results from M2-induced iMG cells showed significant downregulation in genes involved in phagocytic activity in the progressive group. Further, we also found a significant positive correlation between the relative expression of *RNF213* in M2-induced iMG cells and phagocytotic capacity. In addition, qPCR-detected expression of *RNF213* in M2-induced iMG cells in the progressive group was significantly lower, whereas the expression of IL-6, an inflammatory cytokine, was significantly higher. The inflammatory response in MMD leads to hyperplasia of intimal vascular SMCs and angiogenesis because of the proliferation of endothelial cells, which leads to luminal narrowing and collateral vessel formation^39^. Another study suggested that reduced phagocytosis produces high levels of reactive oxygen species and secretion of inflammatory cytokines^40^. There are also reports suggesting that neutrophil-mediated excessive inflammation and oxidative stress in a humanized sickle cell mouse model may cause dysfunction of collateral pathway formation after ischemic injury^41^. These results indicate that inflammatory response may be induced in the progressive group by downregulated expression of *RNF213* in M2 microglia, causing decreased phagocytosis. We speculate that induction of the inflammatory response can lead to hyperplasia of intimal vascular SMCs and proliferation of endothelial cells, causing luminal narrowing/obstruction in major cerebral arteries of patients with MMD (Fig. 15). Whether these pathways are exclusive to MMD is unknown because iMG cells have not been studied in other arterial diseases. However, several papers have reported suggesting microglial activation in cerebral infarction^12,13^, so similar pathways may apply to other arterial diseases. It is expected that studies using iMG cells will validate this in other arterial diseases in the future.

## Limitation

In this study, only adult patients with MMD were included. MMD has a peak incidence in children under 10 years of age^42^; therefore, including younger age groups would be desirable. However, in our current technique, isolation of iMG cells requires at least 20 mL of blood drawing, which could induce hemodynamic stress in children suffering from cerebrovascular impairment. Accordingly, we recruited only adult patients with MMD who can tolerate a large amount of blood sampling. To overcome the limitation, we plan to improve our technique to reduce the amount of blood needed so that pediatric patients with MMD can be recruited in subsequent studies.

The study was conducted with healthy controls who were younger, with more males (*p* = 0.07), and had a lower BMI (*p* < 0.05). All the clinical data including blood samples were obtained from September 2019 to April 2022 at the Department of Neurosurgery, Kyushu University, making population matching difficult. In addition, inflammation parameters were investigated at the mRNA level and not at the protein level. Significantly lower levels of plasma adiponectin, an adipocyte-derived peptide with anti-inflammatory and anti-atherogenic properties, have been reported in patients with ischemic cerebrovascular disease^43^. In the future, after the initial findings of this study are reported, it will be possible to build stronger evidence by increasing the sample size with the help of many participating institutions to create a more matched population. A multicenter study for this purpose will be necessary. Furthermore, the sample size of the RNA-seq cases in this study was small, four cases each. The small sample size restricts drawing conclusive findings, and the present findings are preliminary. In addition, we did not use multiple test corrections to avoid the risk of false negatives. The purpose of this exploratory pilot study was the global analysis of some biomarkers for future validation studies of MMD. Despite the small sample size, we have successfully detected several statistically significant data. Based on the results of this study, future experiments such as Western Blotting (WB), Immunohistochemistry (IHC), Immunocytochemistry (ICC) or Immunofluorescence (IF) are desired to validate the results and follow-up studies with a larger sample size should be conducted to validate these preliminary findings.

No experiments were conducted to investigate the actual mechanism by which microglia cause effects on Moyamoya vessels in this study. If possible, brain sampling from patients with MMD would be a promising approach to overcome the issue; however, such an approach should be limited only to situations where tissue removal is required for diagnosis or treatment. As diagnosis of MMD is confirmed based on radiographical findings and most surgical procedures on patients are performed for bypass surgery, there are almost no situations that involve brain tissue sampling. In addition, although *RNF213* was identified as the causal gene, animal models of MMD have not been established yet. These factors remain unsolved for us, so alternative approaches such as advanced cell culture models reflecting organized angiogenesis^44^ or ex-vivo brain tissue assays^45^ would be required to overcome the limitation. A new animal model of MMD has recently been developed^46^. Since this model requires neurosurgical procedures, it is difficult to investigate the involvement of microglia in the MMD pathology in the present study since activation of microglia by neurosurgical procedures is expected. However, when an animal MMD model that does not require neurosurgical processing is developed, it will be important to investigate the interaction between MMD and M2 microglial angiogenesis.

PCR for inflammatory parameters and angiogenesis in iMG cells overlap in expression in controls and MMD. Thus, M2 microglia might not have contributed to all patients with MMD. One of the pathological hypotheses for MMD suggests that microglia may be involved in MMD pathogenesis. However, microglia are not the only ones involved in the pathogenesis of MMD. The activation of microglia has been suggested to change over time depending on the condition and time of year. Thus, even if they were not activated at this time, they may have been activated at other times. We will continue to collect and analyze iMG cells over time to gain insight into the timing of microglial activation and MMD deactivation.

## Conclusion

Our results based on the original iMG technique suggest that M2 microglia are involved in angiogenesis in MMD. In the progressive Moyamoya state, M2 microglia cells may suppress Moyamoya vessels and be involved in vascular endothelial proliferation. To date, there have been no studies to elucidate the relationship between microglia and MMD. There has been no similar report, so to the best of our knowledge, this is the first study to focus on the biobehavior of microglia in MMD by utilization of our originally developed iMG techniques.

In the future, our approach can facilitate exploring M2 microglial bioactivity in MMD, which may unveil the pathogenesis of MMD and provide novel insights for developing promising interventions early in the progression of the disease.

### Supplementary Information

Supplementary Figures.

Supplementary Tables.

Supplementary Legends.

## Data Availability

The RNA-seq datasets during the current study are available in the National Center for Biotechnology Information Sequence Read Archive under accession no. PRJNA877774.
