# Supplementary material for: Angiogenic and inflammatory responses in human induced microglia-like (iMG) cells from patients with Moyamoya disease
Source: Sci Rep. 2023 Sep 8;13:14842. doi: 10.1038/s41598-023-41456-z (PMC10491754; doi:10.1038/s41598-023-41456-z)
Supplement: Supplementary file 1 — Supplementary Figures. [file 41598_2023_41456_MOESM1_ESM.pdf]

Figure S1

qPCR

RNF213

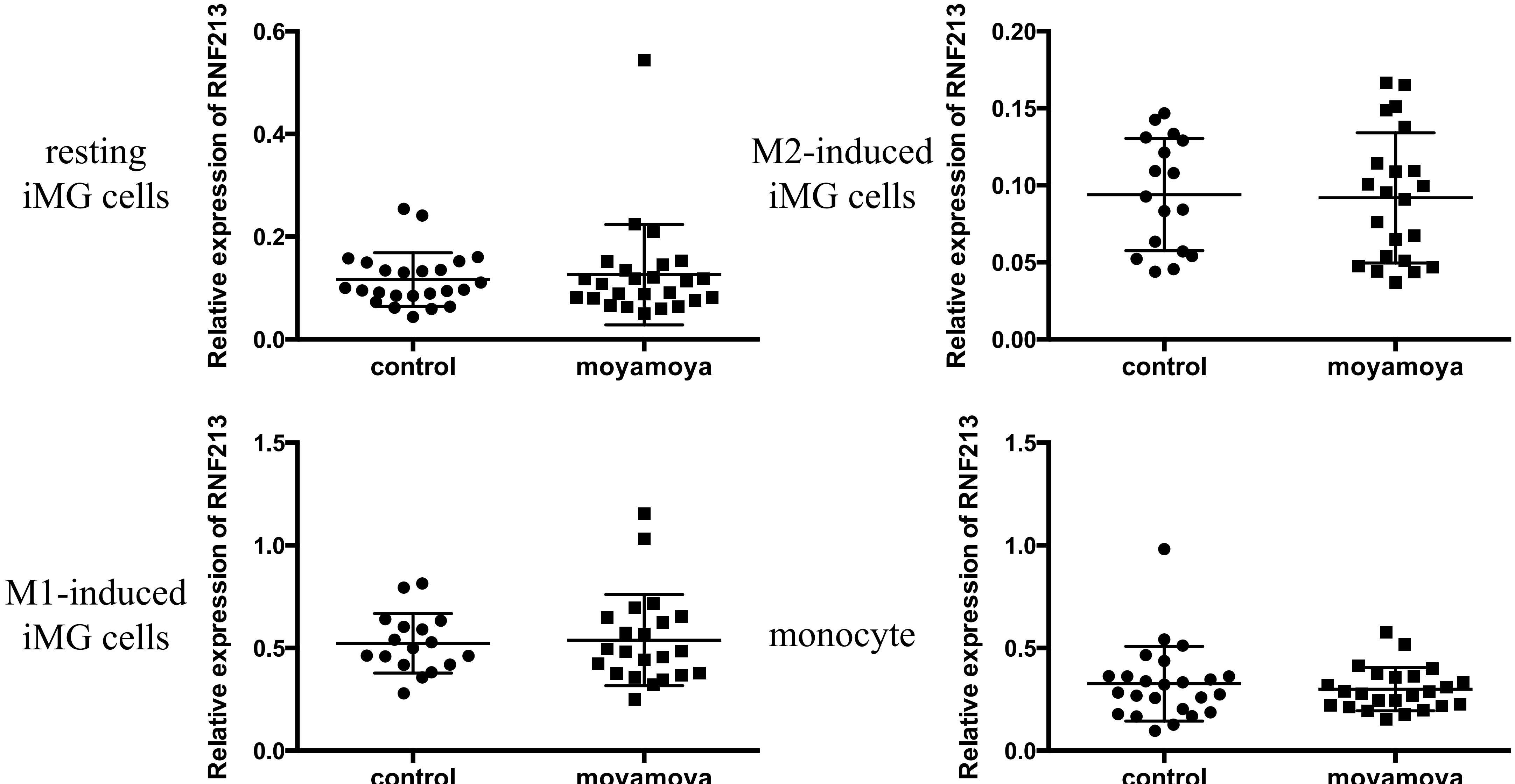

Figure S2

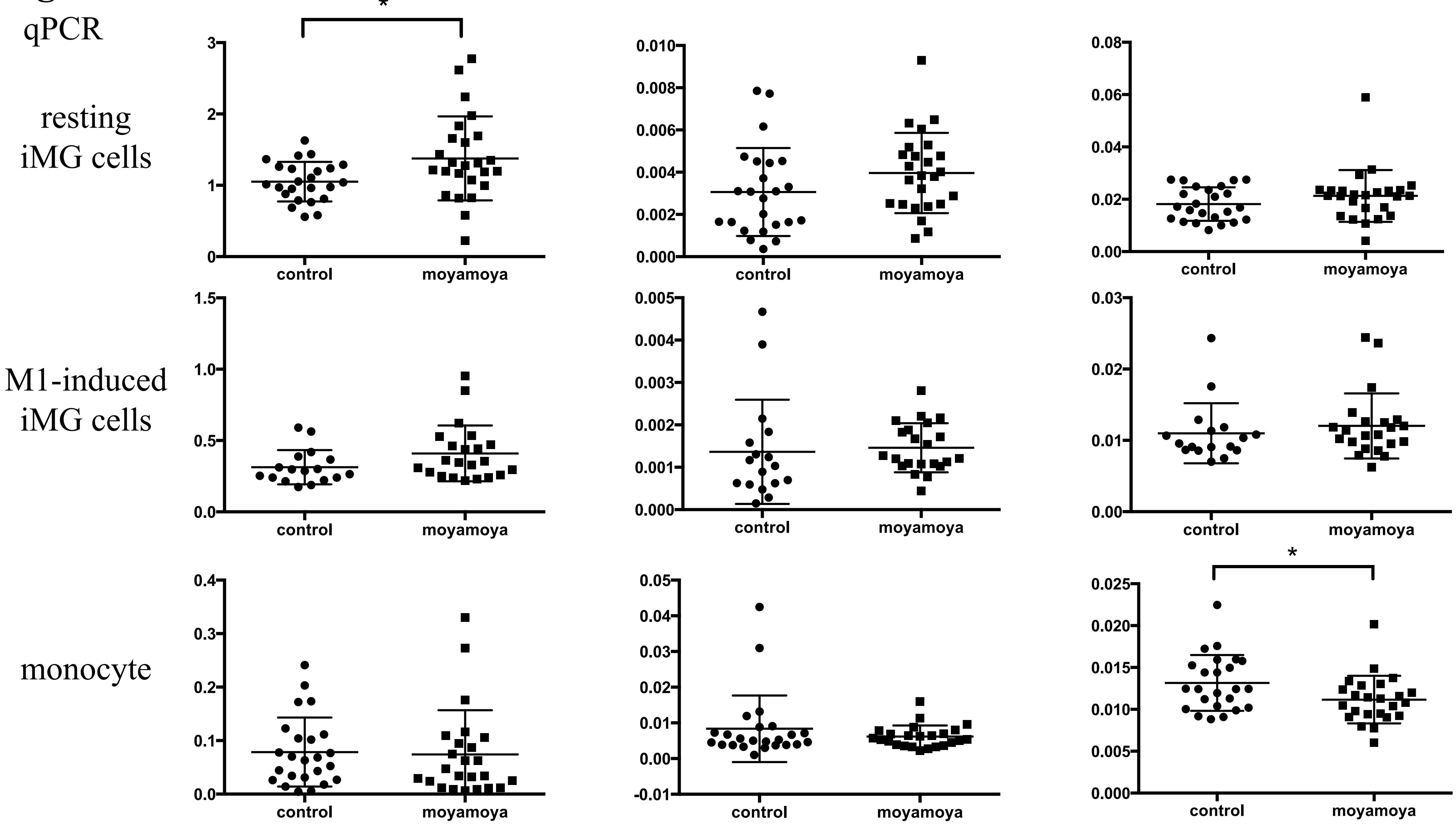
MMP9
VEGFA
TGFB1
qPCR
resting iMG cells
M1-induced iMG cells
monocyte
*
*
control
moyamoya
3
2
1
0
0.010
0.008
0.006
0.004
0.002
0.000
0.08
0.06
0.04
0.02
0.00
1.5
1.0
0.5
0.0
0.005
0.004
0.003
0.002
0.001
0.000
0.03
0.02
0.01
0.00
0.4
0.3
0.2
0.1
0.0
0.05
0.04
0.03
0.02
0.01
0.00
-0.01
0.025
0.020
0.015
0.010
0.005
0.000

Figure S3

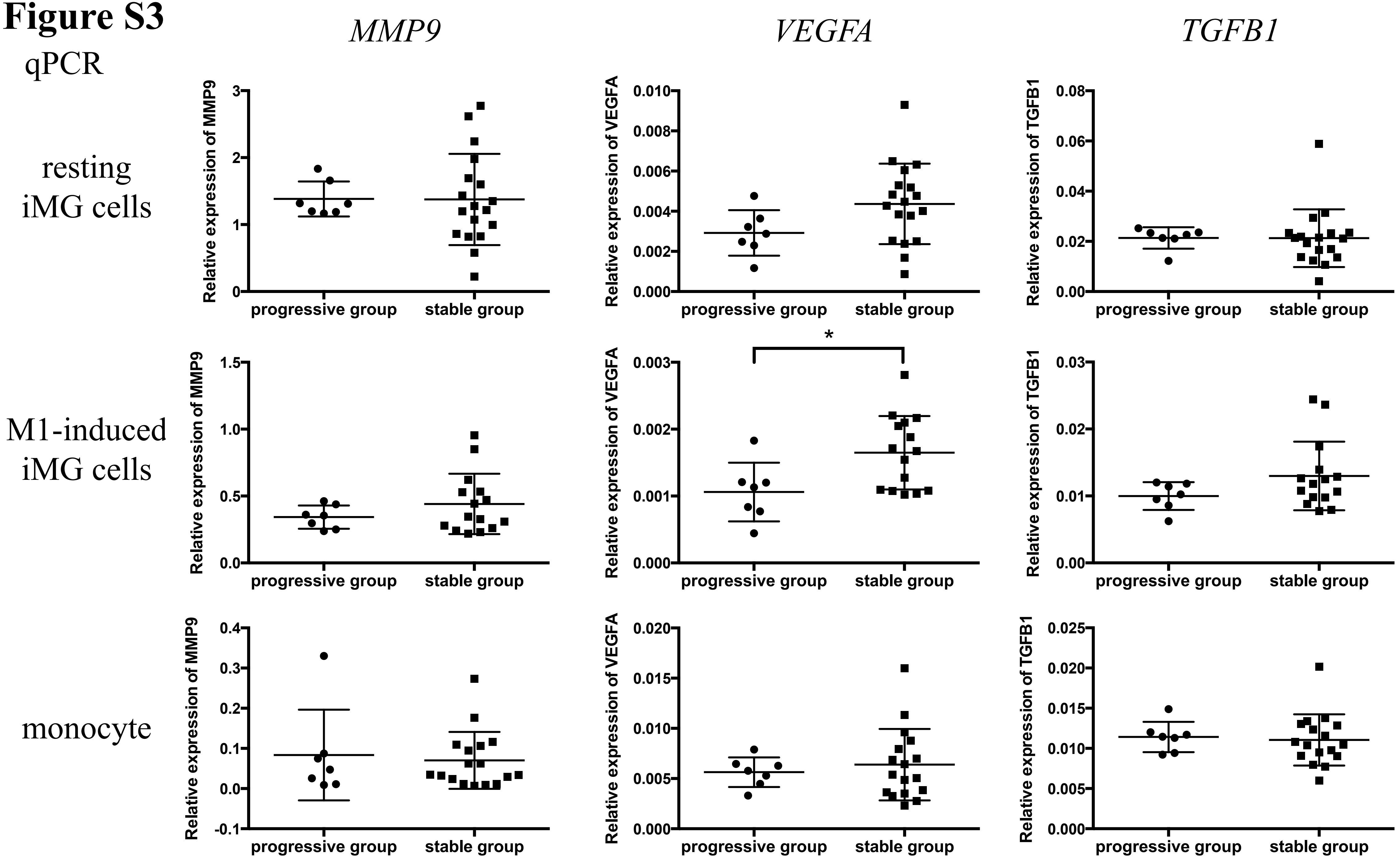
qPCR
MMP9
VEGFA
TGFB1
resting iMG cells
M1-induced iMG cells
monocyte
Relative expression of MMP9
Relative expression of VEGFA
Relative expression of TGFB1
progressive group
stable group
*
0
1
2
3
0.000
0.002
0.004
0.006
0.008
0.010
0.00
0.02
0.04
0.06
0.08
0.0
0.5
1.0
1.5
0.001
0.003
0.01
0.03
-0.1
0.1
0.2
0.3
0.4
0.005
0.015
0.020
0.025
