# Supplementary material for: Angiogenic and inflammatory responses in human induced microglia-like (iMG) cells from patients with Moyamoya disease
Source: Sci Rep. 2023 Sep 8;13:14842. doi: 10.1038/s41598-023-41456-z (PMC10491754; doi:10.1038/s41598-023-41456-z)
Supplement: Supplementary file 2 — Supplementary Tables. [file 41598_2023_41456_MOESM2_ESM.docx]

**Table S1. Primer sequences that were used for qRT-PCR**

| **Gene symbol** | **Forward (5’ to 3’)** | **Reverse (5’ to 3’)** |
| --- | --- | --- |
| IL-6 | GCAGATGAGTACAAAAGTCCTGA | TTCTGTGCCTGCAGCTTC |
| VEGFA | GTACAAGATCCGCAGACGTG | TTCTGTATCAGTCTTTCCTGGTG |
| MMP-9 | ACATCGTCATCCAGTTTGGTG | CGTCGAAATGGCGTCT |
| TGF-β1 | CCGACTACTACGCCAAGGA | GTTCAGGTACCGCTTCTCG |
| RNF213 | AGTACGTCAACCGCTGTCT | GTGGTTCATGACTTTCTGGAGT |
| GAPDH | ACATCGCTCAGACACCATG | TGTAGTTGAGGTCAATGAAGGG |
| B2M | GGACTGGTCTTTCTATCTCTTGT | ACCTCCATGATGCTGCTTAC |

**Table S2. Summary of differential expression analysis using M2-induced iMG cells between moyamoya disease and healthy controls**

| **ID** | **Symbol** | **Expression Mean** | **Expression healthy controls** | **Expression moyamoya disease** | **log2FC** | **lfcSE** | **Stat** | ***p*-value** | **padj** |
| --- | --- | --- | --- | --- | --- | --- | --- | --- | --- |
| ENSG00000237599.9 | TAP2 | 8.539 | 0 | 12.809 | 3.787 | 2.976 | 8.332 | 8.0e-17 | 1.9e-12 |
| ENSG00000237724.5 | HSPA1A | 12.036 | 0 | 18.054 | 4.252 | 3.226 | 8.141 | 3.9e-16 | 4.7e-12 |
| ENSG00000183742.12 | MACC1 | 3.551 | 1.464 | 4.595 | 1.183 | 0.288 | 7.404 | 1.3e-13 | 1.1e-9 |
| ENSG00000119535.17 | CSF3R | 12.266 | 24.992 | 5.903 | -1.913 | 0.274 | -7.069 | 1.6e-12 | 8.1e-9 |
| ENSG00000124006.14 | OBSL1 | 5.596 | 10.987 | 2.9 | -1.62 | 0.243 | -7.059 | 1.7e-12 | 8.1e-9 |
| ENSG00000070669.16 | ASNS | 17.152 | 6.129 | 22.664 | 1.731 | 0.313 | 6.795 | 1.1e-11 | 4.3e-8 |
| ENSG00000005001.9 | PRSS22 | 8.297 | 1.899 | 11.496 | 2.108 | 0.373 | 6.755 | 1.4e-11 | 4.9e-8 |
| ENSG00000106070.18 | GRB10 | 6.541 | 3.419 | 8.103 | 1.043 | 0.219 | 6.539 | 6.2e-11 | 1.9e-7 |
| ENSG00000136010.13 | ALDH1L2 | 8.58 | 3.302 | 11.219 | 1.506 | 0.306 | 6.384 | 1.7e-10 | 4.6e-7 |
| ENSG00000092621.12 | PHGDH | 22.148 | 4.553 | 30.945 | 2.524 | 0.437 | 6.258 | 3.9e-10 | 8.7e-7 |
| ENSG00000120457.11 | KCNJ5 | 0.971 | 0.412 | 1.25 | 0.672 | 0.29 | 6.255 | 4.0e-10 | 8.7e-7 |
| ENSG00000128165.8 | ADM2 | 2.576 | 0.748 | 3.49 | 1.361 | 0.445 | 6.233 | 4.6e-10 | 8.7e-7 |
| ENSG00000135069.13 | PSAT1 | 27.791 | 9.798 | 36.787 | 1.807 | 0.313 | 6.229 | 4.7e-10 | 8.7e-7 |
| ENSG00000154027.18 | AK5 | 42.13 | 14.434 | 55.978 | 1.884 | 0.384 | 6.207 | 5.4e-10 | 9.3e-7 |
| ENSG00000148344.10 | PTGES | 1.868 | 0.184 | 2.711 | 1.648 | 0.635 | 6.071 | 1.3e-9 | 2.0e-6 |
| ENSG00000166448.14 | TMEM130 | 4.517 | 10.127 | 1.713 | -2.036 | 0.508 | -6.05 | 1.4e-9 | 2.2e-6 |
| ENSG00000139793.18 | MBNL2 | 13.577 | 9.399 | 15.667 | 0.68 | 0.127 | 5.684 | 1.3e-8 | 1.9e-5 |
| ENSG00000031698.12 | SARS | 79.815 | 56.226 | 91.609 | 0.694 | 0.125 | 5.616 | 2.0e-8 | 2.5e-5 |
| ENSG00000075213.10 | SEMA3A | 2.935 | 1.75 | 3.527 | 0.719 | 0.241 | 5.616 | 2.0e-8 | 2.5e-5 |
| ENSG00000175183.9 | CSRP2 | 30.191 | 17.692 | 36.44 | 1.002 | 0.18 | 5.6 | 2.1e-8 | 2.6e-5 |
| ENSG00000167992.12 | VWCE | 18.006 | 33.125 | 10.447 | -1.576 | 0.307 | -5.5 | 3.8e-8 | 4.3e-5 |
| ENSG00000140254.12 | DUOXA1 | 4.811 | 10.982 | 1.725 | -2.136 | 0.443 | -5.47 | 4.5e-8 | 4.9e-5 |
| ENSG00000120337.8 | TNFSF18 | 0.497 | 1.465 | 0.014 | -1.282 | 1.226 | -5.458 | 4.8e-8 | 5.0e-5 |
| ENSG00000170324.20 | FRMPD2 | 2.829 | 0.945 | 3.77 | 1.294 | 0.402 | 5.349 | 8.8e-8 | 8.8e-5 |
| ENSG00000166833.19 | NAV2 | 1.959 | 0.58 | 2.648 | 1.207 | 0.432 | 5.218 | 1.8e-7 | 1.7e-4 |
| ENSG00000129451.11 | KLK10 | 0.957 | 0.204 | 1.333 | 0.954 | 0.47 | 5.173 | 2.3e-7 | 2.1e-4 |
| ENSG00000274338.2 | CATSPERB | 0.723 | 0.096 | 1.037 | 0.894 | 0.704 | 5.161 | 2.5e-7 | 2.1e-4 |
| ENSG00000076067.12 | RBMS2 | 24.255 | 39.098 | 16.834 | -1.169 | 0.332 | -5.153 | 2.6e-7 | 2.1e-4 |
| ENSG00000086475.14 | SEPHS1 | 24.193 | 34.681 | 18.949 | -0.839 | 0.145 | -5.152 | 2.6e-7 | 2.1e-4 |
| ENSG00000137193.13 | PIM1 | 56.855 | 13.403 | 78.581 | 2.466 | 0.497 | 5.151 | 2.6e-7 | 2.1e-4 |
| ENSG00000126353.3 | CCR7 | 0.401 | 0 | 0.601 | 0.679 | 1.405 | 5.14 | 2.7e-7 | 2.1e-4 |
| ENSG00000183336.8 | BOLA2 | 9.616 | 7.426 | 10.71 | 0.475 | 0.121 | 5.108 | 3.3e-7 | 2.4e-4 |
| ENSG00000143013.12 | LMO4 | 66.517 | 41.185 | 79.184 | 0.927 | 0.201 | 5.094 | 3.5e-7 | 2.6e-4 |
| ENSG00000006451.7 | RALA | 86.88 | 49.549 | 105.545 | 1.076 | 0.239 | 5.087 | 3.6e-7 | 2.6e-4 |
| ENSG00000106066.14 | CPVL | 45.196 | 73.259 | 31.165 | -1.207 | 0.255 | -5.056 | 4.3e-7 | 2.9e-4 |
| ENSG00000179583.19 | CIITA | 15.837 | 31.721 | 7.895 | -1.879 | 0.423 | -5.007 | 5.5e-7 | 3.6e-4 |
| ENSG00000107317.12 | PTGDS | 504.408 | 981.235 | 265.995 | -1.879 | 0.366 | -5.005 | 5.6e-7 | 3.6e-4 |
| ENSG00000135074.15 | ADAM19 | 0.13 | 0.344 | 0.022 | -0.395 | 0.801 | -4.977 | 6.5e-7 | 4.1e-4 |
| ENSG00000196517.11 | SLC6A9 | 0.51 | 0.083 | 0.723 | 0.67 | 0.602 | 4.965 | 6.9e-7 | 4.2e-4 |
| ENSG00000110497.14 | AMBRA1 | 14.744 | 20.51 | 11.861 | -0.742 | 0.146 | -4.961 | 7.0e-7 | 4.2e-4 |
| ENSG00000164695.4 | CHMP4C | 2.764 | 4.34 | 1.976 | -0.844 | 0.218 | -4.946 | 7.6e-7 | 4.4e-4 |
| ENSG00000120875.8 | DUSP4 | 0.51 | 0.173 | 0.678 | 0.517 | 0.418 | 4.891 | 1.0e-6 | 5.7e-4 |
| ENSG00000133106.14 | EPSTI1 | 6.11 | 11.763 | 3.283 | -1.575 | 0.409 | -4.865 | 1.1e-6 | 6.3e-4 |
| ENSG00000099998.17 | GGT5 | 0.805 | 1.831 | 0.291 | -1.132 | 0.572 | -4.863 | 1.2e-6 | 6.3e-4 |
| ENSG00000184575.11 | XPOT | 38.414 | 26.257 | 44.492 | 0.739 | 0.163 | 4.857 | 1.2e-6 | 6.4e-4 |
| ENSG00000163293.11 | NIPAL1 | 3.538 | 4.874 | 2.87 | -0.602 | 0.265 | -4.835 | 1.3e-6 | 7.0e-4 |
| ENSG00000123360.11 | PDE1B | 5.404 | 7.612 | 4.3 | -0.7 | 0.155 | -4.804 | 1.6e-6 | 7.9e-4 |
| ENSG00000108523.15 | RNF167 | 89.252 | 76.179 | 95.788 | 0.327 | 0.097 | 4.787 | 1.7e-6 | 8.5e-4 |
| ENSG00000137265.14 | IRF4 | 9.756 | 23.455 | 2.907 | -2.646 | 0.459 | -4.765 | 1.9e-6 | 9.2e-4 |
| ENSG00000183486.12 | MX2 | 9.632 | 15.514 | 6.692 | -1.102 | 0.353 | -4.746 | 2.1e-6 | 9.8e-4 |
| ENSG00000100336.17 | APOL4 | 10.979 | 19.264 | 6.837 | -1.371 | 0.334 | -4.745 | 2.1e-6 | 9.8e-4 |
| ENSG00000110077.14 | MS4A6A | 131.988 | 350.05 | 22.958 | -3.873 | 0.841 | -4.735 | 2.2e-6 | 0.001 |
| ENSG00000197046.11 | SIGLEC15 | 32.012 | 15.885 | 40.075 | 1.282 | 0.277 | 4.723 | 2.3e-6 | 0.001 |
| ENSG00000166927.12 | MS4A7 | 100.534 | 164.093 | 68.755 | -1.243 | 0.268 | -4.721 | 2.4e-6 | 0.001 |
| ENSG00000175505.10 | CLCF1 | 4.141 | 1.097 | 5.663 | 1.668 | 0.526 | 4.692 | 2.7e-6 | 0.001 |
| ENSG00000130477.15 | UNC13A | 1.002 | 0.645 | 1.181 | 0.407 | 0.4 | 4.657 | 3.2e-6 | 0.001 |
| ENSG00000185933.6 | CALHM1 | 0.314 | 0.609 | 0.166 | -0.464 | 0.388 | -4.634 | 3.6e-6 | 0.002 |
| ENSG00000198380.12 | GFPT1 | 24.653 | 15.788 | 29.086 | 0.842 | 0.193 | 4.619 | 3.9e-6 | 0.002 |
| ENSG00000137857.17 | DUOX1 | 9.698 | 16.124 | 6.485 | -1.194 | 0.459 | -4.617 | 3.9e-6 | 0.002 |
| ENSG00000060982.14 | BCAT1 | 136.023 | 94.633 | 156.718 | 0.722 | 0.155 | 4.606 | 4.1e-6 | 0.002 |
| ENSG00000259066.5 | AL110118.2 | 1.594 | 0.395 | 2.193 | 1.195 | 0.541 | 4.602 | 4.2e-6 | 0.002 |
| ENSG00000071242.11 | RPS6KA2 | 16.58 | 11.857 | 18.942 | 0.633 | 0.201 | 4.601 | 4.2e-6 | 0.002 |
| ENSG00000106617.13 | PRKAG2 | 53.067 | 41.755 | 58.723 | 0.482 | 0.127 | 4.595 | 4.3e-6 | 0.002 |
| ENSG00000137309.19 | HMGA1 | 116.389 | 63.863 | 142.652 | 1.147 | 0.232 | 4.592 | 4.4e-6 | 0.002 |
| ENSG00000173801.16 | JUP | 18.345 | 26.296 | 14.37 | -0.829 | 0.202 | -4.585 | 4.5e-6 | 0.002 |
| ENSG00000082641.15 | NFE2L1 | 179.681 | 143.286 | 197.878 | 0.463 | 0.104 | 4.579 | 4.7e-6 | 0.002 |
| ENSG00000019582.14 | CD74 | 2318.806 | 4127.028 | 1414.696 | -1.544 | 0.322 | -4.576 | 4.7e-6 | 0.002 |
| ENSG00000139865.16 | TTC6 | 5.226 | 2.508 | 6.585 | 1.113 | 0.316 | 4.574 | 4.8e-6 | 0.002 |
| ENSG00000106069.22 | CHN2 | 14.772 | 21.457 | 11.43 | -0.853 | 0.234 | -4.573 | 4.8e-6 | 0.002 |
| ENSG00000163393.12 | SLC22A15 | 5.5 | 3.357 | 6.571 | 0.797 | 0.244 | 4.573 | 4.8e-6 | 0.002 |
| ENSG00000143479.15 | DYRK3 | 3.193 | 1.565 | 4.006 | 0.965 | 0.304 | 4.541 | 5.6e-6 | 0.002 |
| ENSG00000133056.13 | PIK3C2B | 2.858 | 3.9 | 2.337 | -0.554 | 0.166 | -4.533 | 5.8e-6 | 0.002 |
| ENSG00000280004.1 | AC005086.3 | 18.732 | 0 | 28.097 | 4.863 | 1.293 | 4.53 | 5.9e-6 | 0.002 |
| ENSG00000064989.12 | CALCRL | 1.175 | 1.936 | 0.794 | -0.71 | 0.401 | -4.523 | 6.1e-6 | 0.002 |
| ENSG00000271605.5 | MILR1 | 54.109 | 74.67 | 43.829 | -0.755 | 0.161 | -4.522 | 6.1e-6 | 0.002 |
| ENSG00000187742.14 | SECISBP2 | 17.294 | 13.943 | 18.97 | 0.418 | 0.102 | 4.518 | 6.2e-6 | 0.002 |
| ENSG00000117791.15 | MARC2 | 6.745 | 10.955 | 4.639 | -1.084 | 0.304 | -4.518 | 6.2e-6 | 0.002 |
| ENSG00000153162.8 | BMP6 | 3.841 | 2.185 | 4.668 | 0.832 | 0.248 | 4.513 | 6.4e-6 | 0.002 |
| ENSG00000049239.12 | H6PD | 11.233 | 15.277 | 9.211 | -0.673 | 0.146 | -4.513 | 6.4e-6 | 0.002 |
| ENSG00000178860.8 | MSC | 107.773 | 63.456 | 129.931 | 1.022 | 0.187 | 4.513 | 6.4e-6 | 0.002 |
| ENSG00000089041.16 | P2RX7 | 53.007 | 30.955 | 64.033 | 1.025 | 0.252 | 4.513 | 6.4e-6 | 0.002 |
| ENSG00000101890.4 | GUCY2F | 0.16 | 0.032 | 0.224 | 0.247 | 0.633 | 4.513 | 6.4e-6 | 0.002 |
| ENSG00000174125.7 | TLR1 | 47.173 | 35.866 | 52.826 | 0.546 | 0.096 | 4.506 | 6.6e-6 | 0.002 |
| ENSG00000125746.16 | EML2 | 81.007 | 65.352 | 88.834 | 0.437 | 0.135 | 4.488 | 7.2e-6 | 0.002 |
| ENSG00000113013.12 | HSPA9 | 331.087 | 236.029 | 378.616 | 0.679 | 0.123 | 4.482 | 7.4e-6 | 0.002 |
| ENSG00000153046.17 | CDYL | 23.141 | 19.226 | 25.099 | 0.368 | 0.092 | 4.482 | 7.4e-6 | 0.002 |
| ENSG00000143842.14 | SOX13 | 21.992 | 13.213 | 26.381 | 0.946 | 0.209 | 4.476 | 7.6e-6 | 0.002 |
| ENSG00000176046.8 | NUPR1 | 29.532 | 4.289 | 42.153 | 3.029 | 0.637 | 4.466 | 8.0e-6 | 0.002 |
| ENSG00000010310.8 | GIPR | 6.636 | 1.769 | 9.069 | 1.863 | 0.453 | 4.46 | 8.2e-6 | 0.002 |
| ENSG00000066455.12 | GOLGA5 | 28.875 | 23.086 | 31.769 | 0.444 | 0.111 | 4.459 | 8.2e-6 | 0.002 |
| ENSG00000168209.4 | DDIT4 | 9.631 | 3.693 | 12.601 | 1.535 | 0.405 | 4.459 | 8.2e-6 | 0.002 |
| ENSG00000100889.11 | PCK2 | 136.103 | 101.034 | 153.638 | 0.6 | 0.129 | 4.453 | 8.5e-6 | 0.002 |
| ENSG00000278032.2 | LY6E | 109.473 | 173.264 | 77.577 | -1.149 | 0.255 | -4.447 | 8.7e-6 | 0.002 |
| ENSG00000121966.6 | CXCR4 | 24.269 | 12.517 | 30.144 | 1.204 | 0.289 | 4.441 | 9.0e-6 | 0.002 |
| ENSG00000167077.12 | MEI1 | 42.886 | 18.027 | 55.315 | 1.565 | 0.348 | 4.441 | 9.0e-6 | 0.002 |
| ENSG00000138134.11 | STAMBPL1 | 4.261 | 7.929 | 2.428 | -1.381 | 0.364 | -4.427 | 9.5e-6 | 0.002 |
| ENSG00000178015.4 | GPR150 | 4.616 | 2.362 | 5.742 | 1.004 | 0.295 | 4.426 | 9.6e-6 | 0.002 |
| ENSG00000127995.16 | CASD1 | 3.435 | 3.856 | 3.224 | -0.201 | 0.169 | -4.423 | 9.7e-6 | 0.002 |
| ENSG00000229859.9 | PGA3 | 0.315 | 0.817 | 0.065 | -0.771 | 0.792 | -4.423 | 9.8e-6 | 0.002 |
| ENSG00000101265.15 | RASSF2 | 19.168 | 27.054 | 15.225 | -0.79 | 0.178 | -4.42 | 9.9e-6 | 0.002 |
| ENSG00000107186.16 | MPDZ | 0.105 | 0.233 | 0.041 | -0.245 | 1.112 | -4.417 | 1.0e-5 | 0.002 |
| ENSG00000161011.19 | SQSTM1 | 534.371 | 353.22 | 624.947 | 0.821 | 0.204 | 4.407 | 1.0e-5 | 0.002 |
| ENSG00000148677.6 | ANKRD1 | 0.325 | 0.039 | 0.468 | 0.498 | 0.817 | 4.386 | 1.2e-5 | 0.003 |
| ENSG00000133805.15 | AMPD3 | 67.344 | 48.185 | 76.923 | 0.664 | 0.156 | 4.385 | 1.2e-5 | 0.003 |
| ENSG00000167749.11 | KLK4 | 38.542 | 21.402 | 47.111 | 1.103 | 0.394 | 4.379 | 1.2e-5 | 0.003 |
| ENSG00000236177.9 | HLA-DPA1 | 208.593 | 381.797 | 121.991 | -1.638 | 0.352 | -4.375 | 1.2e-5 | 0.003 |
| ENSG00000186318.16 | BACE1 | 18.424 | 25.03 | 15.122 | -0.691 | 0.184 | -4.372 | 1.2e-5 | 0.003 |
| ENSG00000145860.11 | RNF145 | 76.137 | 51.146 | 88.633 | 0.781 | 0.232 | 4.372 | 1.2e-5 | 0.003 |
| ENSG00000110492.15 | MDK | 19.154 | 9.096 | 24.183 | 1.319 | 0.321 | 4.37 | 1.2e-5 | 0.003 |
| ENSG00000282937.4 | PRSS22 | 5.812 | 1.173 | 8.131 | 2.071 | 0.597 | 4.368 | 1.3e-5 | 0.003 |
| ENSG00000026297.15 | RNASET2 | 136.682 | 175.077 | 117.484 | -0.572 | 0.129 | -4.364 | 1.3e-5 | 0.003 |
| ENSG00000113657.12 | DPYSL3 | 20.54 | 6.22 | 27.7 | 1.991 | 0.443 | 4.363 | 1.3e-5 | 0.003 |
| ENSG00000186481.16 | ANKRD20A5P | 3.183 | 6.825 | 1.363 | -1.728 | 0.604 | -4.36 | 1.3e-5 | 0.003 |
| ENSG00000154134.14 | ROBO3 | 3.677 | 4.755 | 3.138 | -0.476 | 0.193 | -4.344 | 1.4e-5 | 0.003 |
| ENSG00000142798.18 | HSPG2 | 6.722 | 10.736 | 4.715 | -1.038 | 0.267 | -4.343 | 1.4e-5 | 0.003 |
| ENSG00000105281.12 | SLC1A5 | 108.409 | 59.334 | 132.947 | 1.151 | 0.271 | 4.341 | 1.4e-5 | 0.003 |
| ENSG00000206466.10 | GABBR1 | 1.958 | 4.075 | 0.9 | -1.418 | 0.577 | -4.337 | 1.4e-5 | 0.003 |
| ENSG00000024422.11 | EHD2 | 4.097 | 2.672 | 4.81 | 0.662 | 0.449 | 4.337 | 1.4e-5 | 0.003 |
| ENSG00000131845.14 | ZNF304 | 2.297 | 2.929 | 1.98 | -0.399 | 0.13 | -4.336 | 1.4e-5 | 0.003 |
| ENSG00000115211.15 | EIF2B4 | 22.556 | 18.283 | 24.692 | 0.414 | 0.098 | 4.332 | 1.5e-5 | 0.003 |
| ENSG00000142599.17 | RERE | 36.081 | 42.691 | 32.775 | -0.371 | 0.12 | -4.324 | 1.5e-5 | 0.003 |
| ENSG00000173890.16 | GPR160 | 2.262 | 3.629 | 1.578 | -0.845 | 0.31 | -4.316 | 1.6e-5 | 0.003 |
| ENSG00000135778.11 | NTPCR | 21.84 | 29.492 | 18.014 | -0.681 | 0.119 | -4.307 | 1.7e-5 | 0.003 |
| ENSG00000189241.6 | TSPYL1 | 24.673 | 30.162 | 21.929 | -0.443 | 0.097 | -4.302 | 1.7e-5 | 0.003 |
| ENSG00000169908.11 | TM4SF1 | 1.58 | 0.193 | 2.273 | 1.456 | 0.839 | 4.3 | 1.7e-5 | 0.003 |
| ENSG00000164039.14 | BDH2 | 16.098 | 22.845 | 12.725 | -0.797 | 0.185 | -4.295 | 1.7e-5 | 0.003 |
| ENSG00000168495.12 | POLR3D | 14.827 | 11.894 | 16.294 | 0.424 | 0.173 | 4.278 | 1.9e-5 | 0.004 |
| ENSG00000214193.10 | SH3D21 | 9.629 | 4.767 | 12.059 | 1.179 | 0.33 | 4.278 | 1.9e-5 | 0.004 |
| ENSG00000129667.12 | RHBDF2 | 56.805 | 40.588 | 64.913 | 0.664 | 0.184 | 4.274 | 1.9e-5 | 0.004 |
| ENSG00000198795.10 | ZNF521 | 0.783 | 0.142 | 1.104 | 0.882 | 0.568 | 4.271 | 1.9e-5 | 0.004 |
| ENSG00000205336.11 | ADGRG1 | 1.298 | 1.001 | 1.447 | 0.29 | 0.861 | 4.268 | 2.0e-5 | 0.004 |
| ENSG00000175040.5 | CHST2 | 3.334 | 1.138 | 4.432 | 1.345 | 0.458 | 4.266 | 2.0e-5 | 0.004 |
| ENSG00000120262.9 | CCDC170 | 1.398 | 2.82 | 0.687 | -1.179 | 0.531 | -4.264 | 2.0e-5 | 0.004 |
| ENSG00000101266.18 | CSNK2A1 | 63.67 | 57.705 | 66.652 | 0.205 | 0.085 | 4.264 | 2.0e-5 | 0.004 |
| ENSG00000132432.13 | SEC61G | 124.441 | 85.76 | 143.781 | 0.739 | 0.183 | 4.264 | 2.0e-5 | 0.004 |
| ENSG00000106113.18 | CRHR2 | 1.301 | 2.353 | 0.775 | -0.918 | 0.389 | -4.257 | 2.1e-5 | 0.004 |
| ENSG00000003249.13 | DBNDD1 | 0.662 | 1.273 | 0.357 | -0.744 | 0.422 | -4.252 | 2.1e-5 | 0.004 |
| ENSG00000121413.12 | ZSCAN18 | 4.233 | 6.063 | 3.317 | -0.71 | 0.256 | -4.249 | 2.2e-5 | 0.004 |
| ENSG00000110944.8 | IL23A | 1.995 | 0.737 | 2.624 | 1.061 | 0.402 | 4.246 | 2.2e-5 | 0.004 |
| ENSG00000169429.10 | CXCL8 | 89.823 | 16.532 | 126.468 | 2.862 | 0.687 | 4.246 | 2.2e-5 | 0.004 |
| ENSG00000123358.19 | NR4A1 | 1.79 | 3.956 | 0.707 | -1.538 | 0.659 | -4.245 | 2.2e-5 | 0.004 |
| ENSG00000157600.11 | TMEM164 | 29.053 | 29.721 | 28.719 | -0.048 | 0.069 | -4.243 | 2.2e-5 | 0.004 |
| ENSG00000138448.11 | ITGAV | 75.744 | 53.154 | 87.04 | 0.701 | 0.226 | 4.239 | 2.2e-5 | 0.004 |
| ENSG00000124491.15 | F13A1 | 124.831 | 352.007 | 11.244 | -4.85 | 1.148 | -4.238 | 2.3e-5 | 0.004 |
| ENSG00000128965.11 | CHAC1 | 3.033 | 1.039 | 4.03 | 1.303 | 0.461 | 4.23 | 2.3e-5 | 0.004 |
| ENSG00000145604.15 | SKP2 | 2.417 | 3.227 | 2.011 | -0.489 | 0.173 | -4.228 | 2.4e-5 | 0.004 |
| ENSG00000149131.15 | SERPING1 | 22.01 | 33.767 | 16.132 | -1.021 | 0.259 | -4.227 | 2.4e-5 | 0.004 |
| ENSG00000217801.9 | AL390719.1 | 19.176 | 8.762 | 24.383 | 1.379 | 0.386 | 4.222 | 2.4e-5 | 0.004 |
| ENSG00000118777.11 | ABCG2 | 1.688 | 2.971 | 1.047 | -0.956 | 0.354 | -4.214 | 2.5e-5 | 0.004 |
| ENSG00000256235.1 | SMIM3 | 14.361 | 9.747 | 16.669 | 0.717 | 0.189 | 4.213 | 2.5e-5 | 0.004 |
| ENSG00000112096.17 | SOD2 | 539.221 | 276.765 | 670.45 | 1.273 | 0.263 | 4.203 | 2.6e-5 | 0.004 |
| ENSG00000188282.12 | RUFY4 | 1.146 | 2.239 | 0.6 | -1.018 | 0.555 | -4.202 | 2.6e-5 | 0.004 |
| ENSG00000125657.4 | TNFSF9 | 3.99 | 1.882 | 5.043 | 1.068 | 0.346 | 4.199 | 2.7e-5 | 0.004 |
| ENSG00000112139.14 | MDGA1 | 12.143 | 1.109 | 17.66 | 3.145 | 0.704 | 4.183 | 2.9e-5 | 0.004 |
| ENSG00000185745.9 | IFIT1 | 6.357 | 12.559 | 3.256 | -1.672 | 0.429 | -4.183 | 2.9e-5 | 0.004 |
| ENSG00000177675.8 | CD163L1 | 5.544 | 14.805 | 0.913 | -3.046 | 0.959 | -4.18 | 2.9e-5 | 0.004 |
| ENSG00000141756.18 | FKBP10 | 1.808 | 0.278 | 2.573 | 1.484 | 0.741 | 4.179 | 2.9e-5 | 0.004 |
| ENSG00000004809.13 | SLC22A16 | 4.545 | 7.062 | 3.286 | -0.911 | 0.357 | -4.167 | 3.1e-5 | 0.005 |
| ENSG00000131016.16 | AKAP12 | 4.774 | 8.48 | 2.921 | -1.274 | 0.347 | -4.165 | 3.1e-5 | 0.005 |
| ENSG00000204136.10 | GGTA1P | 75.204 | 123.539 | 51.037 | -1.259 | 0.293 | -4.158 | 3.2e-5 | 0.005 |
| ENSG00000132507.17 | EIF5A | 285.765 | 245.413 | 305.941 | 0.317 | 0.089 | 4.154 | 3.3e-5 | 0.005 |
| ENSG00000168748.13 | CA7 | 0.259 | 0.685 | 0.046 | -0.688 | 1.074 | -4.151 | 3.3e-5 | 0.005 |
| ENSG00000099308.10 | MAST3 | 6.851 | 7.72 | 6.417 | -0.234 | 0.111 | -4.148 | 3.4e-5 | 0.005 |
| ENSG00000013375.15 | PGM3 | 55.127 | 49.61 | 57.886 | 0.218 | 0.21 | 4.145 | 3.4e-5 | 0.005 |
| ENSG00000057704.12 | TMCC3 | 7.89 | 4.578 | 9.545 | 0.919 | 0.307 | 4.141 | 3.5e-5 | 0.005 |
| ENSG00000103257.8 | SLC7A5 | 24.31 | 15.59 | 28.669 | 0.839 | 0.21 | 4.14 | 3.5e-5 | 0.005 |
| ENSG00000113739.10 | STC2 | 0.484 | 0.302 | 0.575 | 0.274 | 0.623 | 4.132 | 3.6e-5 | 0.005 |
| ENSG00000188641.13 | DPYD | 29.599 | 39.466 | 24.665 | -0.657 | 0.159 | -4.131 | 3.6e-5 | 0.005 |
| ENSG00000184221.12 | OLIG1 | 0.868 | 0.175 | 1.214 | 0.914 | 0.513 | 4.13 | 3.6e-5 | 0.005 |
| ENSG00000183773.15 | AIFM3 | 3.901 | 5.754 | 2.975 | -0.765 | 0.235 | -4.125 | 3.7e-5 | 0.005 |
| ENSG00000119729.11 | RHOQ | 177.661 | 121.828 | 205.577 | 0.75 | 0.181 | 4.108 | 4.0e-5 | 0.006 |
| ENSG00000011347.9 | SYT7 | 5.677 | 1.967 | 7.532 | 1.524 | 0.446 | 4.106 | 4.0e-5 | 0.006 |
| ENSG00000158301.18 | GPRASP2 | 1.795 | 2.493 | 1.446 | -0.514 | 0.204 | -4.103 | 4.1e-5 | 0.006 |
| ENSG00000186188.10 | FFAR4 | 0.466 | 1.002 | 0.199 | -0.74 | 0.521 | -4.099 | 4.2e-5 | 0.006 |
| ENSG00000167748.10 | KLK1 | 6.898 | 2.381 | 9.157 | 1.587 | 0.483 | 4.099 | 4.2e-5 | 0.006 |
| ENSG00000074842.7 | MYDGF | 161.766 | 132.937 | 176.18 | 0.404 | 0.101 | 4.098 | 4.2e-5 | 0.006 |
| ENSG00000013588.7 | GPRC5A | 0.359 | 0.056 | 0.51 | 0.516 | 0.578 | 4.092 | 4.3e-5 | 0.006 |
| ENSG00000181789.14 | COPG1 | 164.354 | 125.698 | 183.682 | 0.544 | 0.133 | 4.088 | 4.3e-5 | 0.006 |
| ENSG00000260916.7 | CCPG1 | 51.824 | 39.932 | 57.769 | 0.522 | 0.137 | 4.077 | 4.6e-5 | 0.006 |
| ENSG00000081320.10 | STK17B | 3.895 | 5.737 | 2.974 | -0.762 | 0.229 | -4.073 | 4.7e-5 | 0.006 |
| ENSG00000117318.8 | ID3 | 8.605 | 16.075 | 4.87 | -1.54 | 0.376 | -4.068 | 4.7e-5 | 0.006 |
| ENSG00000185436.11 | IFNLR1 | 2.823 | 4.858 | 1.805 | -1.062 | 0.331 | -4.066 | 4.8e-5 | 0.006 |
| ENSG00000175203.15 | DCTN2 | 127.025 | 115.395 | 132.84 | 0.201 | 0.058 | 4.065 | 4.8e-5 | 0.006 |
| ENSG00000206380.11 | C6orf48 | 54.306 | 42.738 | 60.09 | 0.482 | 0.146 | 4.064 | 4.8e-5 | 0.006 |
| ENSG00000056736.9 | IL17RB | 2.774 | 5.583 | 1.37 | -1.474 | 0.51 | -4.063 | 4.8e-5 | 0.006 |
| ENSG00000152558.14 | TMEM123 | 115.072 | 147.678 | 98.77 | -0.576 | 0.138 | -4.062 | 4.9e-5 | 0.006 |
| ENSG00000184985.16 | SORCS2 | 0.092 | 0 | 0.138 | 0.186 | 1.23 | 4.054 | 5.0e-5 | 0.006 |
| ENSG00000110079.16 | MS4A4A | 77.735 | 107.896 | 62.654 | -0.775 | 0.212 | -4.051 | 5.1e-5 | 0.007 |
| ENSG00000171777.15 | RASGRP4 | 2.134 | 4.064 | 1.169 | -1.224 | 0.445 | -4.049 | 5.1e-5 | 0.007 |
| ENSG00000241399.6 | CD302 | 11.913 | 21.069 | 7.335 | -1.405 | 0.36 | -4.045 | 5.2e-5 | 0.007 |
| ENSG00000115295.19 | CLIP4 | 35.498 | 28.233 | 39.13 | 0.457 | 0.135 | 4.039 | 5.4e-5 | 0.007 |
| ENSG00000137411.17 | VARS2 | 1.095 | 0 | 1.643 | 1.402 | 2.405 | 4.037 | 5.4e-5 | 0.007 |
| ENSG00000187735.13 | TCEA1 | 36.888 | 26.756 | 41.954 | 0.63 | 0.138 | 4.036 | 5.4e-5 | 0.007 |
| ENSG00000186340.14 | THBS2 | 0.476 | 0.088 | 0.67 | 0.619 | 0.512 | 4.035 | 5.5e-5 | 0.007 |
| ENSG00000088386.16 | SLC15A1 | 0.295 | 0.487 | 0.2 | -0.31 | 0.319 | -4.028 | 5.6e-5 | 0.007 |
| ENSG00000283486.2 | FAM95C | 2.24 | 4.611 | 1.054 | -1.45 | 0.518 | -4.024 | 5.7e-5 | 0.007 |
| ENSG00000154978.12 | VOPP1 | 94.137 | 80.309 | 101.051 | 0.328 | 0.091 | 4.023 | 5.7e-5 | 0.007 |
| ENSG00000091129.19 | NRCAM | 1.972 | 3.338 | 1.288 | -0.923 | 0.421 | -4.02 | 5.8e-5 | 0.007 |
| ENSG00000203710.11 | CR1 | 13.26 | 18.602 | 10.589 | -0.758 | 0.225 | -4.012 | 6.0e-5 | 0.007 |
| ENSG00000006118.14 | TMEM132A | 41.491 | 21.607 | 51.434 | 1.214 | 0.385 | 4.011 | 6.0e-5 | 0.007 |
| ENSG00000181458.10 | TMEM45A | 5.373 | 3.47 | 6.324 | 0.712 | 0.16 | 4.011 | 6.1e-5 | 0.007 |
| ENSG00000137959.15 | IFI44L | 1.162 | 2.357 | 0.564 | -1.102 | 0.533 | -4.009 | 6.1e-5 | 0.007 |
| ENSG00000010319.6 | SEMA3G | 0.087 | 0.21 | 0.025 | -0.238 | 0.978 | -4.007 | 6.1e-5 | 0.007 |
| ENSG00000000971.15 | CFH | 1.15 | 2.504 | 0.473 | -1.251 | 0.68 | -4.005 | 6.2e-5 | 0.007 |
| ENSG00000128272.14 | ATF4 | 142.176 | 105.827 | 160.35 | 0.595 | 0.154 | 4.003 | 6.2e-5 | 0.007 |
| ENSG00000081665.13 | ZNF506 | 4.777 | 6.325 | 4.003 | -0.55 | 0.185 | -3.999 | 6.4e-5 | 0.007 |
| ENSG00000138449.10 | SLC40A1 | 1.206 | 2.404 | 0.607 | -1.082 | 0.701 | -3.985 | 6.7e-5 | 0.008 |
| ENSG00000096433.10 | ITPR3 | 0.61 | 0.19 | 0.82 | 0.613 | 0.532 | 3.982 | 6.8e-5 | 0.008 |
| ENSG00000197712.11 | FAM114A1 | 11.706 | 7.184 | 13.967 | 0.871 | 0.297 | 3.977 | 7.0e-5 | 0.008 |
| ENSG00000120265.16 | PCMT1 | 73.817 | 58.794 | 81.328 | 0.461 | 0.118 | 3.974 | 7.1e-5 | 0.008 |
| ENSG00000146192.14 | FGD2 | 48.886 | 71.795 | 37.432 | -0.922 | 0.232 | -3.968 | 7.3e-5 | 0.008 |
| ENSG00000122591.11 | FAM126A | 25.589 | 46.194 | 15.287 | -1.535 | 0.365 | -3.966 | 7.3e-5 | 0.008 |
| ENSG00000142405.21 | NLRP12 | 0.987 | 0.535 | 1.213 | 0.528 | 0.307 | 3.964 | 7.4e-5 | 0.008 |
| ENSG00000184005.10 | ST6GALNAC3 | 0.628 | 0.231 | 0.826 | 0.569 | 0.587 | 3.963 | 7.4e-5 | 0.008 |
| ENSG00000025434.18 | NR1H3 | 494.921 | 290.993 | 596.885 | 1.034 | 0.279 | 3.962 | 7.4e-5 | 0.008 |
| ENSG00000109472.13 | CPE | 7.458 | 12.581 | 4.897 | -1.203 | 0.394 | -3.961 | 7.5e-5 | 0.008 |
| ENSG00000080546.13 | SESN1 | 11.65 | 14.616 | 10.167 | -0.484 | 0.145 | -3.96 | 7.5e-5 | 0.008 |
| ENSG00000133059.16 | DSTYK | 4.363 | 5.465 | 3.811 | -0.426 | 0.119 | -3.955 | 7.7e-5 | 0.008 |
| ENSG00000068438.14 | FTSJ1 | 20.757 | 15.328 | 23.472 | 0.584 | 0.142 | 3.955 | 7.7e-5 | 0.008 |
| ENSG00000125648.14 | SLC25A23 | 25.393 | 41.653 | 17.263 | -1.224 | 0.337 | -3.952 | 7.7e-5 | 0.008 |
| ENSG00000173868.11 | PHOSPHO1 | 4.065 | 7.324 | 2.435 | -1.277 | 0.432 | -3.946 | 8.0e-5 | 0.009 |
| ENSG00000143387.12 | CTSK | 626.791 | 315.071 | 782.651 | 1.31 | 0.341 | 3.945 | 8.0e-5 | 0.009 |
| ENSG00000155760.2 | FZD7 | 1.238 | 0.583 | 1.566 | 0.697 | 0.366 | 3.945 | 8.0e-5 | 0.009 |
| ENSG00000179388.8 | EGR3 | 0.669 | 1.497 | 0.255 | -0.992 | 0.863 | -3.94 | 8.1e-5 | 0.009 |
| ENSG00000042286.14 | AIFM2 | 16.555 | 12.573 | 18.545 | 0.526 | 0.145 | 3.936 | 8.3e-5 | 0.009 |
| ENSG00000152270.8 | PDE3B | 14.287 | 19.375 | 11.743 | -0.677 | 0.173 | -3.929 | 8.5e-5 | 0.009 |
| ENSG00000014914.20 | MTMR11 | 0.797 | 0.318 | 1.036 | 0.627 | 0.516 | 3.929 | 8.5e-5 | 0.009 |
| ENSG00000086730.16 | LAT2 | 35.911 | 54.737 | 26.499 | -1.019 | 0.269 | -3.924 | 8.7e-5 | 0.009 |
| ENSG00000130733.10 | YIPF2 | 37.82 | 28.946 | 42.257 | 0.531 | 0.209 | 3.923 | 8.7e-5 | 0.009 |
| ENSG00000145022.4 | TCTA | 18.918 | 14.53 | 21.112 | 0.51 | 0.14 | 3.914 | 9.1e-5 | 0.009 |
| ENSG00000076555.15 | ACACB | 5.266 | 6.432 | 4.683 | -0.387 | 0.157 | -3.91 | 9.2e-5 | 0.009 |
| ENSG00000111860.13 | CEP85L | 2.196 | 2.464 | 2.062 | -0.178 | 0.156 | -3.91 | 9.2e-5 | 0.009 |
| ENSG00000113163.16 | COL4A3BP | 61.488 | 43.816 | 70.324 | 0.67 | 0.192 | 3.91 | 9.2e-5 | 0.009 |
| ENSG00000111181.12 | SLC6A12 | 23.124 | 13.748 | 27.813 | 0.966 | 0.245 | 3.909 | 9.3e-5 | 0.009 |
| ENSG00000157601.13 | MX1 | 20.035 | 40.659 | 9.723 | -1.958 | 0.636 | -3.909 | 9.3e-5 | 0.009 |
| ENSG00000036828.16 | CASR | 0.457 | 0.232 | 0.569 | 0.348 | 0.287 | 3.902 | 9.5e-5 | 0.01 |
| ENSG00000277753.3 | MUC20 | 3.086 | 1.012 | 4.123 | 1.348 | 0.669 | 3.902 | 9.5e-5 | 0.01 |
| ENSG00000181634.7 | TNFSF15 | 31.636 | 6.581 | 44.163 | 2.575 | 0.696 | 3.9 | 9.6e-5 | 0.01 |
| ENSG00000022267.16 | FHL1 | 2.24 | 3.92 | 1.4 | -1.036 | 0.394 | -3.897 | 9.7e-5 | 0.01 |
| ENSG00000168334.8 | XIRP1 | 0.479 | 0.073 | 0.682 | 0.649 | 0.84 | 3.896 | 9.8e-5 | 0.01 |
| ENSG00000160208.12 | RRP1B | 13.188 | 15.679 | 11.942 | -0.366 | 0.089 | -3.892 | 9.9e-5 | 0.01 |
| ENSG00000115919.14 | KYNU | 93.22 | 59.257 | 110.201 | 0.884 | 0.241 | 3.891 | 1.0e-4 | 0.01 |
| ENSG00000182389.19 | CACNB4 | 0.734 | 1.163 | 0.519 | -0.509 | 0.334 | -3.885 | 1.0e-4 | 0.01 |
| ENSG00000117115.12 | PADI2 | 0.512 | 1.187 | 0.175 | -0.896 | 0.724 | -3.881 | 1.0e-4 | 0.01 |
| ENSG00000196967.10 | ZNF585A | 7.959 | 10.648 | 6.615 | -0.613 | 0.114 | -3.872 | 1.1e-4 | 0.011 |
| ENSG00000198796.6 | ALPK2 | 0.082 | 0 | 0.123 | 0.167 | 1.486 | 3.872 | 1.1e-4 | 0.011 |
| ENSG00000173535.14 | TNFRSF10C | 4.704 | 6.712 | 3.699 | -0.715 | 0.284 | -3.871 | 1.1e-4 | 0.011 |
| ENSG00000122733.12 | PHF24 | 0.405 | 0.159 | 0.527 | 0.398 | 0.77 | 3.869 | 1.1e-4 | 0.011 |
| ENSG00000165983.14 | PTER | 13.363 | 10.01 | 15.039 | 0.543 | 0.138 | 3.869 | 1.1e-4 | 0.011 |
| ENSG00000163827.12 | LRRC2 | 0.168 | 0.039 | 0.233 | 0.247 | 0.629 | 3.868 | 1.1e-4 | 0.011 |
| ENSG00000265787.2 | CYP4F35P | 1.052 | 2.27 | 0.443 | -1.181 | 0.585 | -3.866 | 1.1e-4 | 0.011 |
| ENSG00000070366.13 | SMG6 | 15.454 | 17.426 | 14.468 | -0.252 | 0.089 | -3.859 | 1.1e-4 | 0.011 |
| ENSG00000136628.17 | EPRS | 65.103 | 49.827 | 72.741 | 0.537 | 0.113 | 3.859 | 1.1e-4 | 0.011 |
| ENSG00000159479.16 | MED8 | 30.995 | 26.546 | 33.22 | 0.313 | 0.103 | 3.858 | 1.1e-4 | 0.011 |
| ENSG00000256043.2 | CTSO | 11.164 | 13.972 | 9.76 | -0.477 | 0.122 | -3.851 | 1.2e-4 | 0.011 |
| ENSG00000158286.12 | RNF207 | 12.067 | 4.265 | 15.969 | 1.688 | 0.32 | 3.846 | 1.2e-4 | 0.011 |
| ENSG00000153064.11 | BANK1 | 0.506 | 1.011 | 0.254 | -0.681 | 0.601 | -3.843 | 1.2e-4 | 0.011 |
| ENSG00000241106.6 | HLA-DOB | 1 | 0.023 | 1.488 | 1.281 | 1.584 | 3.838 | 1.2e-4 | 0.012 |
| ENSG00000137965.10 | IFI44 | 24.898 | 38.673 | 18.01 | -1.061 | 0.281 | -3.835 | 1.3e-4 | 0.012 |
| ENSG00000165195.15 | PIGA | 8.482 | 4.146 | 10.65 | 1.179 | 0.165 | 3.833 | 1.3e-4 | 0.012 |
| ENSG00000183283.15 | DAZAP2 | 223.766 | 263.483 | 203.907 | -0.368 | 0.084 | -3.827 | 1.3e-4 | 0.012 |
| ENSG00000183010.16 | PYCR1 | 3.469 | 1.718 | 4.345 | 0.975 | 0.356 | 3.827 | 1.3e-4 | 0.012 |
| ENSG00000100453.12 | GZMB | 5.219 | 0.347 | 7.656 | 2.684 | 1.134 | 3.821 | 1.3e-4 | 0.012 |
| ENSG00000064655.18 | EYA2 | 1.135 | 2.495 | 0.455 | -1.264 | 0.639 | -3.82 | 1.3e-4 | 0.012 |
| ENSG00000175352.10 | NRIP3 | 28.92 | 20.031 | 33.364 | 0.708 | 0.188 | 3.817 | 1.4e-4 | 0.012 |
| ENSG00000150977.10 | RILPL2 | 10.511 | 6.879 | 12.328 | 0.758 | 0.228 | 3.815 | 1.4e-4 | 0.012 |
| ENSG00000047457.13 | CP | 13.663 | 3.208 | 18.89 | 2.241 | 0.673 | 3.81 | 1.4e-4 | 0.012 |
| ENSG00000115520.8 | COQ10B | 19.195 | 15.75 | 20.918 | 0.388 | 0.112 | 3.809 | 1.4e-4 | 0.013 |
| ENSG00000051382.8 | PIK3CB | 33.736 | 23.319 | 38.945 | 0.716 | 0.197 | 3.807 | 1.4e-4 | 0.013 |
| ENSG00000141753.6 | IGFBP4 | 5.348 | 12.993 | 1.525 | -2.47 | 0.789 | -3.804 | 1.4e-4 | 0.013 |
| ENSG00000134369.15 | NAV1 | 6.44 | 4.594 | 7.362 | 0.58 | 0.2 | 3.804 | 1.4e-4 | 0.013 |
| ENSG00000113407.13 | TARS | 62.948 | 51.618 | 68.613 | 0.404 | 0.106 | 3.799 | 1.5e-4 | 0.013 |
| ENSG00000178038.16 | ALS2CL | 1.081 | 0.48 | 1.382 | 0.687 | 0.362 | 3.797 | 1.5e-4 | 0.013 |
| ENSG00000111335.12 | OAS2 | 17.222 | 22.281 | 14.693 | -0.569 | 0.202 | -3.796 | 1.5e-4 | 0.013 |
| ENSG00000005486.16 | RHBDD2 | 53.417 | 85.309 | 37.47 | -1.166 | 0.228 | -3.792 | 1.5e-4 | 0.013 |
| ENSG00000153485.5 | TMEM251 | 75.031 | 58.066 | 83.514 | 0.517 | 0.15 | 3.792 | 1.5e-4 | 0.013 |
| ENSG00000147883.10 | CDKN2B | 8.175 | 4.304 | 10.111 | 1.067 | 0.298 | 3.79 | 1.5e-4 | 0.013 |
| ENSG00000132530.16 | XAF1 | 21.092 | 34.139 | 14.569 | -1.174 | 0.276 | -3.785 | 1.5e-4 | 0.013 |
| ENSG00000167232.13 | ZNF91 | 3.887 | 5.185 | 3.238 | -0.545 | 0.143 | -3.777 | 1.6e-4 | 0.014 |
| ENSG00000125841.12 | NRSN2 | 9.761 | 7.062 | 11.111 | 0.587 | 0.197 | 3.774 | 1.6e-4 | 0.014 |
| ENSG00000197506.7 | SLC28A3 | 144.29 | 100.275 | 166.298 | 0.724 | 0.224 | 3.774 | 1.6e-4 | 0.014 |
| ENSG00000167468.16 | GPX4 | 559.348 | 436.356 | 620.844 | 0.508 | 0.135 | 3.772 | 1.6e-4 | 0.014 |
| ENSG00000124370.10 | MCEE | 11.88 | 8.43 | 13.604 | 0.631 | 0.164 | 3.772 | 1.6e-4 | 0.014 |
| ENSG00000185624.14 | P4HB | 965.042 | 697.761 | 1098.682 | 0.654 | 0.163 | 3.77 | 1.6e-4 | 0.014 |
| ENSG00000106178.6 | CCL24 | 7.513 | 17.918 | 2.311 | -2.515 | 0.778 | -3.767 | 1.7e-4 | 0.014 |
| ENSG00000165915.13 | SLC39A13 | 109.839 | 84.846 | 122.336 | 0.523 | 0.13 | 3.766 | 1.7e-4 | 0.014 |
| ENSG00000106245.10 | BUD31 | 189.83 | 158.995 | 205.247 | 0.366 | 0.094 | 3.764 | 1.7e-4 | 0.014 |
| ENSG00000171649.11 | ZIK1 | 1.009 | 1.312 | 0.857 | -0.316 | 0.259 | -3.763 | 1.7e-4 | 0.014 |
| ENSG00000188211.8 | NCR3LG1 | 3.283 | 4.693 | 2.578 | -0.67 | 0.219 | -3.763 | 1.7e-4 | 0.014 |
| ENSG00000172548.14 | NIPAL4 | 2.538 | 0.291 | 3.662 | 1.853 | 1.061 | 3.76 | 1.7e-4 | 0.014 |
| ENSG00000048545.13 | GUCA1A | 4.696 | 9.155 | 2.467 | -1.55 | 0.487 | -3.754 | 1.7e-4 | 0.014 |
| ENSG00000010327.10 | STAB1 | 53.57 | 142.214 | 9.248 | -3.805 | 0.952 | -3.753 | 1.7e-4 | 0.014 |
| ENSG00000136141.14 | LRCH1 | 29.921 | 22.527 | 33.619 | 0.557 | 0.155 | 3.752 | 1.8e-4 | 0.014 |
| ENSG00000077232.17 | DNAJC10 | 69.973 | 59.855 | 75.032 | 0.321 | 0.109 | 3.752 | 1.8e-4 | 0.014 |
| ENSG00000134291.11 | TMEM106C | 7.602 | 9.324 | 6.74 | -0.415 | 0.156 | -3.751 | 1.8e-4 | 0.014 |
| ENSG00000184988.8 | TMEM106A | 16.586 | 24.222 | 12.768 | -0.873 | 0.206 | -3.75 | 1.8e-4 | 0.014 |
| ENSG00000101197.12 | BIRC7 | 2.073 | 0.46 | 2.879 | 1.41 | 0.659 | 3.75 | 1.8e-4 | 0.014 |
| ENSG00000185950.8 | IRS2 | 5.525 | 3.662 | 6.456 | 0.677 | 0.225 | 3.749 | 1.8e-4 | 0.014 |
| ENSG00000104419.14 | NDRG1 | 75.416 | 46.637 | 89.806 | 0.931 | 0.325 | 3.742 | 1.8e-4 | 0.015 |
| ENSG00000135378.3 | PRRG4 | 1.045 | 1.647 | 0.743 | -0.602 | 0.293 | -3.73 | 1.9e-4 | 0.015 |
| ENSG00000157388.16 | CACNA1D | 1.517 | 2.658 | 0.946 | -0.911 | 0.347 | -3.727 | 1.9e-4 | 0.015 |
| ENSG00000167797.7 | CDK2AP2 | 51.196 | 33.384 | 60.101 | 0.829 | 0.236 | 3.725 | 2.0e-4 | 0.016 |
| ENSG00000135587.8 | SMPD2 | 19.531 | 12.482 | 23.056 | 0.835 | 0.23 | 3.721 | 2.0e-4 | 0.016 |
| ENSG00000275395.5 | FCGBP | 18.494 | 50.116 | 2.684 | -3.795 | 1.099 | -3.714 | 2.0e-4 | 0.016 |
| ENSG00000171812.12 | COL8A2 | 24.266 | 12.678 | 30.061 | 1.183 | 0.343 | 3.711 | 2.1e-4 | 0.016 |
| ENSG00000184897.5 | H1FX | 68.174 | 43.771 | 80.375 | 0.862 | 0.243 | 3.707 | 2.1e-4 | 0.016 |
| ENSG00000151135.9 | TMEM263 | 11.893 | 7.523 | 14.077 | 0.823 | 0.23 | 3.704 | 2.1e-4 | 0.017 |
| ENSG00000131508.15 | UBE2D2 | 203.684 | 177.888 | 216.582 | 0.283 | 0.098 | 3.699 | 2.2e-4 | 0.017 |
| ENSG00000105135.15 | ILVBL | 42.796 | 36.14 | 46.124 | 0.343 | 0.109 | 3.693 | 2.2e-4 | 0.017 |
| ENSG00000163634.11 | THOC7 | 31.127 | 38.717 | 27.332 | -0.487 | 0.105 | -3.692 | 2.2e-4 | 0.017 |
| ENSG00000133134.11 | BEX2 | 4.415 | 1.418 | 5.914 | 1.515 | 0.572 | 3.689 | 2.3e-4 | 0.017 |
| ENSG00000132821.11 | VSTM2L | 1.423 | 2.732 | 0.768 | -1.078 | 0.612 | -3.689 | 2.3e-4 | 0.017 |
| ENSG00000134363.11 | FST | 1.295 | 2.641 | 0.622 | -1.166 | 0.56 | -3.687 | 2.3e-4 | 0.017 |
| ENSG00000187626.8 | ZKSCAN4 | 2.51 | 3.594 | 1.968 | -0.63 | 0.22 | -3.68 | 2.3e-4 | 0.018 |
| ENSG00000162493.16 | PDPN | 63.197 | 32.798 | 78.397 | 1.232 | 0.326 | 3.679 | 2.3e-4 | 0.018 |
| ENSG00000120885.21 | CLU | 302.826 | 590.898 | 158.789 | -1.889 | 0.505 | -3.676 | 2.4e-4 | 0.018 |
| ENSG00000162817.6 | C1orf115 | 2.671 | 5.118 | 1.447 | -1.322 | 0.47 | -3.676 | 2.4e-4 | 0.018 |
| ENSG00000163395.16 | IGFN1 | 2.404 | 0.698 | 3.257 | 1.326 | 0.675 | 3.673 | 2.4e-4 | 0.018 |
| ENSG00000172432.18 | GTPBP2 | 53.053 | 38.116 | 60.521 | 0.653 | 0.181 | 3.672 | 2.4e-4 | 0.018 |
| ENSG00000088992.17 | TESC | 5.422 | 3.148 | 6.559 | 0.866 | 0.276 | 3.672 | 2.4e-4 | 0.018 |
| ENSG00000105519.15 | CAPS | 13.085 | 8.538 | 15.358 | 0.778 | 0.257 | 3.666 | 2.5e-4 | 0.018 |
| ENSG00000156804.7 | FBXO32 | 7.884 | 5.022 | 9.315 | 0.776 | 0.29 | 3.666 | 2.5e-4 | 0.018 |
| ENSG00000123342.15 | MMP19 | 76.369 | 45.962 | 91.573 | 0.979 | 0.283 | 3.666 | 2.5e-4 | 0.018 |
| ENSG00000243742.5 | RPLP0P2 | 31.17 | 17.008 | 38.251 | 1.124 | 0.373 | 3.663 | 2.5e-4 | 0.018 |
| ENSG00000167680.15 | SEMA6B | 6.023 | 3.697 | 7.186 | 0.801 | 0.29 | 3.661 | 2.5e-4 | 0.018 |
| ENSG00000119508.17 | NR4A3 | 9.019 | 15.455 | 5.801 | -1.275 | 0.364 | -3.661 | 2.5e-4 | 0.018 |
| ENSG00000110237.4 | ARHGEF17 | 4.128 | 5.653 | 3.365 | -0.608 | 0.184 | -3.656 | 2.6e-4 | 0.019 |
| ENSG00000225151.10 | GOLGA2P7 | 1.16 | 0.566 | 1.457 | 0.65 | 0.442 | 3.656 | 2.6e-4 | 0.019 |
| ENSG00000105514.7 | RAB3D | 1.632 | 2.594 | 1.151 | -0.741 | 0.282 | -3.654 | 2.6e-4 | 0.019 |
| ENSG00000175279.21 | CENPS | 10.082 | 9.428 | 10.408 | 0.13 | 0.192 | 3.647 | 2.6e-4 | 0.019 |
| ENSG00000224557.7 | HLA-DPB2 | 0.171 | 0.453 | 0.03 | -0.497 | 1.074 | -3.647 | 2.7e-4 | 0.019 |
| ENSG00000136877.14 | FPGS | 73.54 | 58.668 | 80.977 | 0.458 | 0.113 | 3.64 | 2.7e-4 | 0.02 |
| ENSG00000054690.13 | PLEKHH1 | 1.757 | 2.45 | 1.41 | -0.518 | 0.279 | -3.64 | 2.7e-4 | 0.02 |
| ENSG00000135597.18 | REPS1 | 18.611 | 14.625 | 20.605 | 0.467 | 0.17 | 3.631 | 2.8e-4 | 0.02 |
| ENSG00000135114.12 | OASL | 0.389 | 0.672 | 0.248 | -0.423 | 0.529 | -3.628 | 2.9e-4 | 0.02 |
| ENSG00000167371.18 | PRRT2 | 1.279 | 0.808 | 1.515 | 0.476 | 0.284 | 3.628 | 2.9e-4 | 0.02 |
| ENSG00000110057.7 | UNC93B1 | 77.021 | 92.915 | 69.075 | -0.422 | 0.116 | -3.626 | 2.9e-4 | 0.021 |
| ENSG00000169245.5 | CXCL10 | 0.502 | 1.37 | 0.068 | -1.15 | 1.21 | -3.625 | 2.9e-4 | 0.021 |
| ENSG00000115594.11 | IL1R1 | 12.71 | 19.59 | 9.27 | -1.003 | 0.276 | -3.624 | 2.9e-4 | 0.021 |
| ENSG00000182224.11 | CYB5D1 | 4.622 | 3.66 | 5.104 | 0.389 | 0.164 | 3.623 | 2.9e-4 | 0.021 |
| ENSG00000131459.12 | GFPT2 | 0.761 | 0.089 | 1.097 | 0.945 | 0.879 | 3.62 | 2.9e-4 | 0.021 |
| ENSG00000137713.15 | PPP2R1B | 11.652 | 10.245 | 12.355 | 0.248 | 0.119 | -3.62 | 3.0e-4 | 0.021 |
| ENSG00000162413.16 | KLHL21 | 22.533 | 15.109 | 26.245 | 0.758 | 0.236 | 3.619 | 3.0e-4 | 0.021 |
| ENSG00000134247.9 | PTGFRN | 7.926 | 11.341 | 6.219 | -0.774 | 0.231 | -3.609 | 3.1e-4 | 0.021 |
| ENSG00000153132.12 | CLGN | 0.723 | 0.325 | 0.923 | 0.537 | 0.508 | 3.608 | 3.1e-4 | 0.022 |
| ENSG00000106105.13 | GARS | 209.222 | 156.407 | 235.63 | 0.588 | 0.155 | 3.604 | 3.1e-4 | 0.022 |
| ENSG00000155011.8 | DKK2 | 5.043 | 8.449 | 3.34 | -1.123 | 0.362 | -3.603 | 3.1e-4 | 0.022 |
| ENSG00000103707.9 | MTFMT | 12.982 | 16.391 | 11.278 | -0.502 | 0.16 | -3.603 | 3.1e-4 | 0.022 |
| ENSG00000141258.12 | SGSM2 | 63.038 | 69.32 | 59.897 | -0.208 | 0.101 | -3.602 | 3.2e-4 | 0.022 |
| ENSG00000158106.13 | RHPN1 | 3.509 | 5.891 | 2.317 | -1.055 | 0.351 | -3.6 | 3.2e-4 | 0.022 |
| ENSG00000145911.5 | N4BP3 | 0.793 | 0.346 | 1.017 | 0.583 | 0.437 | 3.599 | 3.2e-4 | 0.022 |
| ENSG00000102580.14 | DNAJC3 | 30.547 | 24.309 | 33.667 | 0.454 | 0.14 | 3.597 | 3.2e-4 | 0.022 |
| ENSG00000143226.13 | FCGR2A | 219.316 | 394.223 | 131.863 | -1.573 | 0.369 | -3.595 | 3.2e-4 | 0.022 |
| ENSG00000065911.11 | MTHFD2 | 111.732 | 80.86 | 127.168 | 0.647 | 0.185 | 3.592 | 3.3e-4 | 0.022 |
| ENSG00000198586.13 | TLK1 | 35.127 | 27.464 | 38.958 | 0.489 | 0.153 | 3.582 | 3.4e-4 | 0.023 |
| ENSG00000074935.13 | TUBE1 | 7.027 | 6.05 | 7.516 | 0.272 | 0.176 | 3.577 | 3.5e-4 | 0.023 |
| ENSG00000133612.18 | AGAP3 | 129.709 | 107.986 | 140.57 | 0.377 | 0.128 | 3.576 | 3.5e-4 | 0.024 |
| ENSG00000204438.10 | GPANK1 | 7.938 | 6.085 | 8.864 | 0.478 | 0.282 | 3.575 | 3.5e-4 | 0.024 |
| ENSG00000166681.13 | BEX3 | 82.241 | 108.774 | 68.975 | -0.65 | 0.173 | -3.566 | 3.6e-4 | 0.024 |
| ENSG00000125304.9 | TM9SF2 | 113.533 | 97.977 | 121.311 | 0.305 | 0.095 | 3.564 | 3.7e-4 | 0.024 |
| ENSG00000122986.13 | HVCN1 | 50.322 | 70.417 | 40.274 | -0.791 | 0.207 | -3.564 | 3.7e-4 | 0.024 |
| ENSG00000010278.13 | CD9 | 483.041 | 369.389 | 539.867 | 0.546 | 0.166 | 3.563 | 3.7e-4 | 0.024 |
| ENSG00000198933.9 | TBKBP1 | 15.034 | 11.838 | 16.632 | 0.458 | 0.169 | 3.559 | 3.7e-4 | 0.025 |
| ENSG00000148516.21 | ZEB1 | 0.989 | 0.938 | 1.014 | 0.056 | 0.304 | 3.558 | 3.7e-4 | 0.025 |
| ENSG00000075651.15 | PLD1 | 21.065 | 14.707 | 24.244 | 0.685 | 0.178 | 3.555 | 3.8e-4 | 0.025 |
| ENSG00000074590.13 | NUAK1 | 0.274 | 0.054 | 0.384 | 0.394 | 1.405 | 3.551 | 3.8e-4 | 0.025 |
| ENSG00000010818.9 | HIVEP2 | 1.724 | 1.23 | 1.97 | 0.413 | 0.295 | 3.551 | 3.8e-4 | 0.025 |
| ENSG00000111711.9 | GOLT1B | 40.603 | 30.235 | 45.787 | 0.583 | 0.162 | 3.549 | 3.9e-4 | 0.025 |
| ENSG00000077150.18 | NFKB2 | 63.706 | 49.002 | 71.057 | 0.527 | 0.172 | 3.548 | 3.9e-4 | 0.025 |
| ENSG00000185262.8 | UBALD2 | 227.741 | 179.358 | 251.933 | 0.488 | 0.186 | 3.547 | 3.9e-4 | 0.025 |
| ENSG00000116698.21 | SMG7 | 33.238 | 39.59 | 30.062 | -0.386 | 0.072 | -3.547 | 3.9e-4 | 0.025 |
| ENSG00000111912.19 | NCOA7 | 15.508 | 21.125 | 12.699 | -0.692 | 0.196 | -3.547 | 3.9e-4 | 0.025 |
| ENSG00000183196.8 | CHST6 | 0.256 | 0.131 | 0.319 | 0.221 | 0.275 | 3.547 | 3.9e-4 | 0.025 |
| ENSG00000134830.5 | C5AR2 | 7.45 | 4.597 | 8.876 | 0.819 | 0.288 | 3.546 | 3.9e-4 | 0.025 |
| ENSG00000074706.13 | IPCEF1 | 1.197 | 2.548 | 0.522 | -1.221 | 0.694 | -3.544 | 3.9e-4 | 0.025 |
| ENSG00000124613.8 | ZNF391 | 0.131 | 0.323 | 0.034 | -0.356 | 0.76 | -3.544 | 3.9e-4 | 0.025 |
| ENSG00000250644.3 | AC068580.4 | 22.558 | 15.081 | 26.296 | 0.763 | 0.257 | 3.543 | 4.0e-4 | 0.025 |
| ENSG00000069493.14 | CLEC2D | 27.957 | 16.99 | 33.44 | 0.937 | 0.277 | 3.542 | 4.0e-4 | 0.025 |
| ENSG00000110514.19 | MADD | 144.858 | 65.303 | 184.636 | 1.485 | 0.281 | 3.54 | 4.0e-4 | 0.025 |
| ENSG00000165457.13 | FOLR2 | 30.849 | 76.777 | 7.886 | -3.13 | 0.923 | -3.538 | 4.0e-4 | 0.025 |
| ENSG00000167110.17 | GOLGA2 | 62.577 | 50.366 | 68.683 | 0.44 | 0.179 | 3.536 | 4.1e-4 | 0.025 |
| ENSG00000108829.9 | LRRC59 | 61.2 | 48.594 | 67.503 | 0.466 | 0.14 | 3.536 | 4.1e-4 | 0.025 |
| ENSG00000116128.10 | BCL9 | 1.265 | 1.799 | 0.998 | -0.486 | 0.296 | -3.536 | 4.1e-4 | 0.025 |
| ENSG00000136811.16 | ODF2 | 9.417 | 12.603 | 7.824 | -0.624 | 0.164 | -3.529 | 4.2e-4 | 0.026 |
| ENSG00000143126.7 | CELSR2 | 0.662 | 0.952 | 0.517 | -0.364 | 0.311 | -3.527 | 4.2e-4 | 0.026 |
| ENSG00000152147.10 | GEMIN6 | 7.324 | 9.452 | 6.259 | -0.526 | 0.121 | -3.526 | 4.2e-4 | 0.026 |
| ENSG00000278458.4 | KANSL1 | 5.27 | 6.443 | 4.683 | -0.389 | 0.663 | -3.524 | 4.3e-4 | 0.026 |
| ENSG00000179918.18 | SEPHS2 | 90.583 | 76.834 | 97.458 | 0.339 | 0.107 | 3.523 | 4.3e-4 | 0.026 |
| ENSG00000074416.13 | MGLL | 88.164 | 59.705 | 102.393 | 0.768 | 0.223 | 3.523 | 4.3e-4 | 0.026 |
| ENSG00000128590.4 | DNAJB9 | 39.705 | 31.278 | 43.919 | 0.477 | 0.17 | 3.52 | 4.3e-4 | 0.026 |
| ENSG00000248769.1 | AC139495.2 | 2.114 | 4.11 | 1.116 | -1.272 | 0.514 | -3.52 | 4.3e-4 | 0.026 |
| ENSG00000168374.10 | ARF4 | 172.721 | 114.752 | 201.706 | 0.808 | 0.267 | 3.52 | 4.3e-4 | 0.026 |
| ENSG00000074695.5 | LMAN1 | 29.652 | 22.819 | 33.069 | 0.516 | 0.129 | 3.52 | 4.3e-4 | 0.026 |
| ENSG00000187601.4 | MAGEH1 | 4.714 | 2.438 | 5.851 | 0.995 | 0.367 | 3.515 | 4.4e-4 | 0.027 |
| ENSG00000160796.16 | NBEAL2 | 29.526 | 22.597 | 32.991 | 0.527 | 0.171 | 3.513 | 4.4e-4 | 0.027 |
| ENSG00000177383.4 | MAGEF1 | 5.668 | 3.644 | 6.681 | 0.726 | 0.256 | 3.512 | 4.4e-4 | 0.027 |
| ENSG00000125977.6 | EIF2S2 | 47.12 | 41.96 | 49.7 | 0.239 | 0.078 | 3.51 | 4.5e-4 | 0.027 |
| ENSG00000133639.4 | BTG1 | 25.685 | 14.12 | 31.468 | 1.103 | 0.27 | 3.51 | 4.5e-4 | 0.027 |
| ENSG00000166130.14 | IKBIP | 17.643 | 12.844 | 20.043 | 0.604 | 0.216 | 3.509 | 4.5e-4 | 0.027 |
| ENSG00000163071.10 | SPATA18 | 12.416 | 15.647 | 10.8 | -0.497 | 0.233 | -3.506 | 4.5e-4 | 0.027 |
| ENSG00000168890.13 | TMEM150A | 17.605 | 20.698 | 16.058 | -0.347 | 0.092 | -3.503 | 4.6e-4 | 0.028 |
| ENSG00000103496.14 | STX4 | 177.054 | 150.442 | 190.359 | 0.338 | 0.111 | 3.502 | 4.6e-4 | 0.028 |
| ENSG00000103942.12 | HOMER2 | 14.075 | 24.633 | 8.797 | -1.388 | 0.446 | -3.501 | 4.6e-4 | 0.028 |
| ENSG00000196639.6 | HRH1 | 3.929 | 7.903 | 1.942 | -1.598 | 0.61 | -3.501 | 4.6e-4 | 0.028 |
| ENSG00000134294.13 | SLC38A2 | 112.464 | 64.662 | 136.365 | 1.065 | 0.283 | 3.499 | 4.7e-4 | 0.028 |
| ENSG00000172164.13 | SNTB1 | 37.755 | 24.888 | 44.188 | 0.804 | 0.232 | 3.499 | 4.7e-4 | 0.028 |
| ENSG00000120899.17 | PTK2B | 35.089 | 27.073 | 39.097 | 0.514 | 0.15 | 3.495 | 4.7e-4 | 0.028 |
| ENSG00000103034.14 | NDRG4 | 9.252 | 3.98 | 11.888 | 1.372 | 0.515 | 3.495 | 4.7e-4 | 0.028 |
| ENSG00000157240.3 | FZD1 | 3.656 | 4.686 | 3.141 | -0.457 | 0.154 | -3.494 | 4.8e-4 | 0.028 |
| ENSG00000126709.14 | IFI6 | 409.298 | 672.22 | 277.836 | -1.272 | 0.358 | -3.493 | 4.8e-4 | 0.028 |
| ENSG00000124357.12 | NAGK | 301.058 | 245.584 | 328.795 | 0.419 | 0.137 | 3.493 | 4.8e-4 | 0.028 |
| ENSG00000235941.5 | HSPA1A | 55.008 | 86.621 | 39.202 | -1.124 | 0.326 | -3.491 | 4.8e-4 | 0.028 |
| ENSG00000171940.13 | ZNF217 | 13.283 | 16.953 | 11.447 | -0.528 | 0.154 | -3.488 | 4.9e-4 | 0.028 |
| ENSG00000284727.1 | AC116562.4 | 0.872 | 1.29 | 0.663 | -0.462 | 0.266 | -3.486 | 4.9e-4 | 0.028 |
| ENSG00000229156.1 | CR769776.2 | 16.631 | 34.115 | 7.889 | -1.982 | 0.581 | -3.486 | 4.9e-4 | 0.028 |
| ENSG00000039560.13 | RAI14 | 77.396 | 34.726 | 98.731 | 1.481 | 0.333 | 3.483 | 5.0e-4 | 0.029 |
| ENSG00000133069.15 | TMCC2 | 1.903 | 3.142 | 1.284 | -0.859 | 0.363 | -3.481 | 5.0e-4 | 0.029 |
| ENSG00000119326.14 | CTNNAL1 | 50.233 | 72.654 | 39.023 | -0.88 | 0.241 | -3.478 | 5.0e-4 | 0.029 |
| ENSG00000114737.15 | CISH | 55.894 | 75.223 | 46.229 | -0.691 | 0.186 | -3.478 | 5.1e-4 | 0.029 |
| ENSG00000123700.4 | KCNJ2 | 4.312 | 6.347 | 3.295 | -0.774 | 0.275 | -3.476 | 5.1e-4 | 0.029 |
| ENSG00000055332.16 | EIF2AK2 | 9.166 | 10.924 | 8.287 | -0.361 | 0.144 | -3.475 | 5.1e-4 | 0.029 |
| ENSG00000064886.13 | CHI3L2 | 9.163 | 15.639 | 5.926 | -1.265 | 0.418 | -3.474 | 5.1e-4 | 0.029 |
| ENSG00000090861.15 | AARS | 51.441 | 40.916 | 56.704 | 0.461 | 0.137 | 3.472 | 5.2e-4 | 0.029 |
| ENSG00000100221.10 | JOSD1 | 39.887 | 32.614 | 43.524 | 0.406 | 0.123 | 3.47 | 5.2e-4 | 0.029 |
| ENSG00000170836.11 | PPM1D | 4.955 | 6.543 | 4.161 | -0.547 | 0.134 | -3.469 | 5.2e-4 | 0.03 |
| ENSG00000267228.7 | AC012254.2 | 16.758 | 9.242 | 20.516 | 1.071 | 0.304 | 3.468 | 5.2e-4 | 0.03 |
| ENSG00000149115.13 | TNKS1BP1 | 53.106 | 45.423 | 56.947 | 0.32 | 0.13 | 3.466 | 5.3e-4 | 0.03 |
| ENSG00000196460.13 | RFX8 | 1.804 | 0.598 | 2.406 | 1.092 | 0.476 | 3.466 | 5.3e-4 | 0.03 |
| ENSG00000157500.10 | APPL1 | 12.755 | 15.655 | 11.305 | -0.437 | 0.135 | -3.465 | 5.3e-4 | 0.03 |
| ENSG00000182326.14 | C1S | 5.978 | 4.189 | 6.872 | 0.601 | 0.266 | 3.464 | 5.3e-4 | 0.03 |
| ENSG00000107147.12 | KCNT1 | 0.241 | 0.135 | 0.294 | 0.19 | 0.414 | 3.464 | 5.3e-4 | 0.03 |
| ENSG00000185787.14 | MORF4L1 | 267.129 | 234.834 | 283.276 | 0.27 | 0.07 | 3.464 | 5.3e-4 | 0.03 |
| ENSG00000155304.5 | HSPA13 | 11.037 | 7.156 | 12.977 | 0.777 | 0.236 | 3.462 | 5.4e-4 | 0.03 |
| ENSG00000148700.14 | ADD3 | 35.284 | 50.321 | 27.766 | -0.835 | 0.277 | -3.459 | 5.4e-4 | 0.03 |
| ENSG00000186907.7 | RTN4RL2 | 7.98 | 4.323 | 9.809 | 1.022 | 0.367 | 3.459 | 5.4e-4 | 0.03 |
| ENSG00000163563.7 | MNDA | 88.558 | 127.886 | 68.894 | -0.883 | 0.24 | -3.458 | 5.4e-4 | 0.03 |
| ENSG00000184432.9 | COPB2 | 94.334 | 79.705 | 101.649 | 0.347 | 0.104 | 3.457 | 5.5e-4 | 0.03 |
| ENSG00000139514.12 | SLC7A1 | 14.016 | 10.08 | 15.983 | 0.616 | 0.199 | 3.453 | 5.5e-4 | 0.03 |
| ENSG00000123636.17 | BAZ2B | 21.777 | 27.415 | 18.957 | -0.51 | 0.125 | -3.448 | 5.6e-4 | 0.031 |
| ENSG00000128016.5 | ZFP36 | 46.981 | 72.697 | 34.123 | -1.069 | 0.276 | -3.447 | 5.7e-4 | 0.031 |
| ENSG00000141574.7 | SECTM1 | 2.497 | 5.522 | 0.985 | -1.716 | 0.717 | -3.445 | 5.7e-4 | 0.031 |
| ENSG00000125730.16 | C3 | 243.356 | 188.697 | 270.686 | 0.518 | 0.239 | 3.441 | 5.8e-4 | 0.031 |
| ENSG00000090615.14 | GOLGA3 | 43.895 | 31.806 | 49.94 | 0.635 | 0.138 | 3.441 | 5.8e-4 | 0.031 |
| ENSG00000122218.14 | COPA | 100.009 | 88.563 | 105.732 | 0.253 | 0.096 | 3.438 | 5.9e-4 | 0.032 |
| ENSG00000115155.17 | OTOF | 1.386 | 2.833 | 0.663 | -1.205 | 0.622 | -3.436 | 5.9e-4 | 0.032 |
| ENSG00000112679.14 | DUSP22 | 29.838 | 37.43 | 26.042 | -0.507 | 0.15 | -3.436 | 5.9e-4 | 0.032 |
| ENSG00000100242.15 | SUN2 | 77.788 | 63.112 | 85.127 | 0.426 | 0.155 | 3.434 | 5.9e-4 | 0.032 |
| ENSG00000184515.10 | BEX5 | 0.187 | 0 | 0.28 | 0.357 | 1.387 | 3.432 | 6.0e-4 | 0.032 |
| ENSG00000158485.10 | CD1B | 8.904 | 15.893 | 5.41 | -1.398 | 0.437 | -3.432 | 6.0e-4 | 0.032 |
| ENSG00000050730.15 | TNIP3 | 1.436 | 0.484 | 1.912 | 0.973 | 0.522 | 3.431 | 6.0e-4 | 0.032 |
| ENSG00000184702.19 | SEPT5 | 1.698 | 2.685 | 1.205 | -0.741 | 0.311 | -3.429 | 6.1e-4 | 0.032 |
| ENSG00000159733.13 | ZFYVE28 | 21.517 | 16.642 | 23.954 | 0.5 | 0.195 | 3.427 | 6.1e-4 | 0.032 |
| ENSG00000148154.9 | UGCG | 33.659 | 21.028 | 39.974 | 0.895 | 0.285 | 3.425 | 6.1e-4 | 0.032 |
| ENSG00000185338.5 | SOCS1 | 24.698 | 35.425 | 19.335 | -0.841 | 0.236 | -3.425 | 6.1e-4 | 0.032 |
| ENSG00000166341.7 | DCHS1 | 1.242 | 0.498 | 1.613 | 0.802 | 0.492 | 3.425 | 6.2e-4 | 0.032 |
| ENSG00000111879.18 | FAM184A | 0.612 | 0.149 | 0.843 | 0.681 | 0.743 | 3.424 | 6.2e-4 | 0.032 |
| ENSG00000179542.15 | SLITRK4 | 1.781 | 1.217 | 2.062 | 0.466 | 0.258 | 3.417 | 6.3e-4 | 0.033 |
| ENSG00000146416.18 | AIG1 | 6 | 9.587 | 4.207 | -1.024 | 0.392 | -3.417 | 6.3e-4 | 0.033 |
| ENSG00000118113.11 | MMP8 | 1.852 | 0.74 | 2.408 | 0.97 | 0.529 | 3.416 | 6.4e-4 | 0.033 |
| ENSG00000244242.1 | IFITM10 | 3.612 | 0.71 | 5.063 | 1.826 | 0.431 | 3.413 | 6.4e-4 | 0.034 |
| ENSG00000184584.12 | TMEM173 | 4.567 | 7.768 | 2.967 | -1.144 | 0.446 | -3.409 | 6.5e-4 | 0.034 |
| ENSG00000135525.18 | MAP7 | 7.72 | 5.446 | 8.857 | 0.613 | 0.211 | 3.408 | 6.5e-4 | 0.034 |
| ENSG00000114423.19 | CBLB | 37.863 | 32.454 | 40.568 | 0.313 | 0.142 | 3.408 | 6.6e-4 | 0.034 |
| ENSG00000164970.14 | FAM219A | 7.535 | 5.821 | 8.392 | 0.461 | 0.152 | 3.407 | 6.6e-4 | 0.034 |
| ENSG00000106785.14 | TRIM14 | 13.514 | 18.041 | 11.251 | -0.636 | 0.201 | -3.402 | 6.7e-4 | 0.035 |
| ENSG00000152484.13 | USP12 | 46.595 | 32.353 | 53.716 | 0.714 | 0.234 | 3.4 | 6.7e-4 | 0.035 |
| ENSG00000178082.6 | TWF1P1 | 0.943 | 0.451 | 1.189 | 0.593 | 0.429 | 3.399 | 6.8e-4 | 0.035 |
| ENSG00000177409.11 | SAMD9L | 14.944 | 19.384 | 12.723 | -0.571 | 0.183 | -3.399 | 6.8e-4 | 0.035 |
| ENSG00000171791.12 | BCL2 | 5.081 | 4.199 | 5.522 | 0.327 | 0.208 | 3.398 | 6.8e-4 | 0.035 |
| ENSG00000106638.15 | TBL2 | 27.195 | 20.696 | 30.445 | 0.535 | 0.196 | 3.397 | 6.8e-4 | 0.035 |
| ENSG00000134278.15 | SPIRE1 | 31.243 | 25.664 | 34.033 | 0.394 | 0.125 | 3.393 | 6.9e-4 | 0.035 |
| ENSG00000096872.15 | IFT74 | 5.189 | 6.262 | 4.653 | -0.361 | 0.154 | -3.392 | 6.9e-4 | 0.035 |
| ENSG00000163377.15 | FAM19A4 | 0.32 | 0.058 | 0.451 | 0.455 | 0.858 | 3.392 | 6.9e-4 | 0.035 |
| ENSG00000134028.14 | ADAMDEC1 | 35.016 | 12.569 | 46.239 | 1.8 | 0.575 | 3.39 | 7.0e-4 | 0.035 |
| ENSG00000157870.14 | FAM213B | 98.475 | 69.463 | 112.981 | 0.694 | 0.194 | 3.39 | 7.0e-4 | 0.035 |
| ENSG00000183615.5 | FAM167B | 0.269 | 0 | 0.403 | 0.489 | 1.611 | 3.388 | 7.0e-4 | 0.035 |
| ENSG00000152782.16 | PANK1 | 1.081 | 1.582 | 0.831 | -0.496 | 0.316 | -3.387 | 7.1e-4 | 0.035 |
| ENSG00000167642.12 | SPINT2 | 239.268 | 327.964 | 194.92 | -0.748 | 0.198 | -3.387 | 7.1e-4 | 0.035 |
| ENSG00000107201.9 | DDX58 | 4.996 | 6.37 | 4.309 | -0.473 | 0.152 | -3.386 | 7.1e-4 | 0.035 |
| ENSG00000274746.4 | ZNF100 | 1.284 | 1.703 | 1.074 | -0.382 | 0.189 | -3.385 | 7.1e-4 | 0.036 |
| ENSG00000047230.14 | CTPS2 | 2.455 | 2.748 | 2.308 | -0.18 | 0.16 | -3.383 | 7.2e-4 | 0.036 |
| ENSG00000067606.16 | PRKCZ | 1.021 | 1.261 | 0.902 | -0.25 | 0.385 | -3.382 | 7.2e-4 | 0.036 |
| ENSG00000205413.7 | SAMD9 | 3.307 | 4.133 | 2.894 | -0.398 | 0.142 | -3.382 | 7.2e-4 | 0.036 |
| ENSG00000064703.11 | DDX20 | 5.419 | 6.555 | 4.851 | -0.369 | 0.122 | -3.378 | 7.3e-4 | 0.036 |
| ENSG00000055163.19 | CYFIP2 | 8.183 | 11.493 | 6.529 | -0.731 | 0.218 | -3.378 | 7.3e-4 | 0.036 |
| ENSG00000136240.9 | KDELR2 | 77.347 | 57.382 | 87.33 | 0.597 | 0.198 | 3.378 | 7.3e-4 | 0.036 |
| ENSG00000163939.18 | PBRM1 | 23.879 | 19.784 | 25.926 | 0.374 | 0.128 | 3.374 | 7.4e-4 | 0.036 |
| ENSG00000108679.12 | LGALS3BP | 203.809 | 296.091 | 157.668 | -0.905 | 0.264 | -3.374 | 7.4e-4 | 0.036 |
| ENSG00000132274.15 | TRIM22 | 50.604 | 66.705 | 42.553 | -0.636 | 0.188 | -3.374 | 7.4e-4 | 0.036 |
| ENSG00000065809.13 | FAM107B | 41.873 | 32.071 | 46.775 | 0.531 | 0.195 | 3.373 | 7.4e-4 | 0.036 |
| ENSG00000102554.13 | KLF5 | 1.584 | 0.551 | 2.1 | 0.999 | 0.304 | 3.37 | 7.5e-4 | 0.037 |
| ENSG00000120318.15 | ARAP3 | 6.896 | 4.103 | 8.292 | 0.865 | 0.286 | 3.368 | 7.6e-4 | 0.037 |
| ENSG00000039068.18 | CDH1 | 1.081 | 2.361 | 0.44 | -1.223 | 0.521 | -3.364 | 7.7e-4 | 0.037 |
| ENSG00000091513.15 | TF | 0.809 | 0.669 | 0.878 | 0.171 | 0.17 | 3.362 | 7.7e-4 | 0.037 |
| ENSG00000167524.14 | SGK494 | 19.127 | 28.277 | 14.552 | -0.913 | 0.261 | -3.362 | 7.7e-4 | 0.037 |
| ENSG00000130303.12 | BST2 | 171.765 | 222.364 | 146.466 | -0.599 | 0.172 | -3.361 | 7.8e-4 | 0.038 |
| ENSG00000172292.14 | CERS6 | 9.336 | 11.567 | 8.22 | -0.447 | 0.139 | -3.359 | 7.8e-4 | 0.038 |
| ENSG00000170382.11 | LRRN2 | 0.198 | 0.093 | 0.25 | 0.193 | 0.466 | 3.357 | 7.9e-4 | 0.038 |
| ENSG00000164182.10 | NDUFAF2 | 13.805 | 11.149 | 15.133 | 0.409 | 0.164 | 3.357 | 7.9e-4 | 0.038 |
| ENSG00000212719.10 | C17orf51 | 2.825 | 2.566 | 2.954 | 0.149 | 0.245 | 3.353 | 8.0e-4 | 0.038 |
| ENSG00000130300.8 | PLVAP | 0.047 | 0.129 | 0.006 | -0.167 | 1.264 | -3.35 | 8.1e-4 | 0.039 |
| ENSG00000165548.10 | TMEM63C | 1.315 | 2.44 | 0.753 | -0.973 | 0.509 | -3.345 | 8.2e-4 | 0.039 |
| ENSG00000099251.14 | HSD17B7P2 | 1.928 | 2.994 | 1.395 | -0.738 | 0.322 | -3.345 | 8.2e-4 | 0.039 |
| ENSG00000152380.9 | FAM151B | 5.286 | 3.673 | 6.093 | 0.602 | 0.21 | 3.343 | 8.3e-4 | 0.04 |
| ENSG00000150681.9 | RGS18 | 1.64 | 3.176 | 0.871 | -1.158 | 0.52 | -3.342 | 8.3e-4 | 0.04 |
| ENSG00000081189.15 | MEF2C | 8.776 | 13.741 | 6.294 | -1.015 | 0.271 | -3.34 | 8.4e-4 | 0.04 |
| ENSG00000275026.1 | GXYLT1P4 | 2.831 | 6.505 | 0.994 | -1.912 | 0.774 | -3.34 | 8.4e-4 | 0.04 |
| ENSG00000106603.18 | COA1 | 23.508 | 31.319 | 19.602 | -0.65 | 0.11 | -3.338 | 8.4e-4 | 0.04 |
| ENSG00000186063.12 | AIDA | 33.387 | 28.133 | 36.013 | 0.345 | 0.125 | 3.337 | 8.5e-4 | 0.04 |
| ENSG00000123836.14 | PFKFB2 | 10.047 | 12.11 | 9.015 | -0.388 | 0.186 | -3.336 | 8.5e-4 | 0.04 |
| ENSG00000255508.7 | AP002990.1 | 5.917 | 4.195 | 6.777 | 0.582 | 0.237 | 3.333 | 8.6e-4 | 0.04 |
| ENSG00000277104.4 | TADA2A | 3.53 | 2.078 | 4.256 | 0.772 | 0.563 | 3.333 | 8.6e-4 | 0.04 |
| ENSG00000236515.3 | ZBTB9 | 5.737 | 4.296 | 6.457 | 0.494 | 0.182 | 3.333 | 8.6e-4 | 0.04 |
| ENSG00000175197.12 | DDIT3 | 58.345 | 37.01 | 69.013 | 0.881 | 0.269 | 3.332 | 8.6e-4 | 0.04 |
| ENSG00000125733.17 | TRIP10 | 45.055 | 29.523 | 52.822 | 0.818 | 0.237 | 3.332 | 8.6e-4 | 0.04 |
| ENSG00000206172.8 | HBA1 | 1.286 | 3.32 | 0.268 | -1.768 | 1.051 | -3.329 | 8.7e-4 | 0.04 |
| ENSG00000173110.7 | HSPA6 | 7.003 | 12.911 | 4.049 | -1.462 | 0.489 | -3.329 | 8.7e-4 | 0.04 |
| ENSG00000224103.7 | HLA-DPA1 | 211.147 | 400.374 | 116.534 | -1.772 | 0.734 | -3.329 | 8.7e-4 | 0.04 |
| ENSG00000167701.13 | GPT | 2.268 | 3.383 | 1.71 | -0.693 | 0.23 | -3.328 | 8.8e-4 | 0.04 |
| ENSG00000106537.7 | TSPAN13 | 6.287 | 3.774 | 7.544 | 0.84 | 0.3 | 3.327 | 8.8e-4 | 0.04 |
| ENSG00000150991.14 | UBC | 1074.454 | 917.76 | 1152.801 | 0.329 | 0.112 | 3.327 | 8.8e-4 | 0.04 |
| ENSG00000010322.15 | NISCH | 14.417 | 20.325 | 11.463 | -0.775 | 0.211 | -3.322 | 8.9e-4 | 0.041 |
| ENSG00000196588.15 | MKL1 | 29.68 | 36.69 | 26.175 | -0.472 | 0.169 | -3.32 | 9.0e-4 | 0.041 |
| ENSG00000130368.5 | MAS1 | 0.645 | 0.361 | 0.787 | 0.393 | 0.345 | 3.317 | 9.1e-4 | 0.042 |
| ENSG00000188613.6 | NANOS1 | 11.909 | 6.418 | 14.655 | 1.078 | 0.387 | 3.316 | 9.1e-4 | 0.042 |
| ENSG00000266028.7 | SRGAP2 | 24.432 | 30.924 | 21.186 | -0.525 | 0.154 | -3.315 | 9.2e-4 | 0.042 |
| ENSG00000161835.10 | GRASP | 3.025 | 2.35 | 3.363 | 0.381 | 0.527 | 3.312 | 9.3e-4 | 0.042 |
| ENSG00000037757.13 | MRI1 | 8.43 | 11.672 | 6.809 | -0.698 | 0.182 | -3.312 | 9.3e-4 | 0.042 |
| ENSG00000103275.19 | UBE2I | 113.878 | 101.963 | 119.836 | 0.231 | 0.075 | 3.311 | 9.3e-4 | 0.042 |
| ENSG00000152207.7 | CYSLTR2 | 0.587 | 1.098 | 0.332 | -0.656 | 0.555 | -3.31 | 9.3e-4 | 0.042 |
| ENSG00000112715.21 | VEGFA | 13.297 | 9.816 | 15.038 | 0.568 | 0.166 | 3.307 | 9.4e-4 | 0.043 |
| ENSG00000147854.16 | UHRF2 | 23.55 | 16.4 | 27.125 | 0.693 | 0.176 | 3.306 | 9.5e-4 | 0.043 |
| ENSG00000008853.16 | RHOBTB2 | 16.771 | 21.299 | 14.507 | -0.524 | 0.175 | -3.305 | 9.5e-4 | 0.043 |
| ENSG00000051108.14 | HERPUD1 | 206.537 | 139.542 | 240.034 | 0.778 | 0.224 | 3.303 | 9.6e-4 | 0.043 |
| ENSG00000145088.8 | EAF2 | 3.698 | 2.468 | 4.313 | 0.615 | 0.248 | 3.302 | 9.6e-4 | 0.043 |
| ENSG00000160746.12 | ANO10 | 34.963 | 30.956 | 36.967 | 0.249 | 0.145 | 3.301 | 9.6e-4 | 0.043 |
| ENSG00000197361.7 | FBXL22 | 0.337 | 0.2 | 0.405 | 0.227 | 0.582 | 3.301 | 9.6e-4 | 0.043 |
| ENSG00000214274.9 | ANG | 4.088 | 4.136 | 4.064 | -0.02 | 0.475 | 3.299 | 9.7e-4 | 0.043 |
| ENSG00000140030.5 | GPR65 | 3.151 | 4.442 | 2.505 | -0.635 | 0.238 | -3.299 | 9.7e-4 | 0.043 |
| ENSG00000197128.11 | ZNF772 | 2.284 | 2.862 | 1.994 | -0.367 | 0.182 | -3.299 | 9.7e-4 | 0.043 |
| ENSG00000123562.16 | MORF4L2 | 171.041 | 135.643 | 188.741 | 0.474 | 0.134 | 3.299 | 9.7e-4 | 0.043 |
| ENSG00000148450.12 | MSRB2 | 3.296 | 5.042 | 2.423 | -0.82 | 0.273 | -3.296 | 9.8e-4 | 0.043 |
| ENSG00000277518.3 | MUC6 | 0.081 | 0.175 | 0.034 | -0.184 | 0.709 | -3.295 | 9.8e-4 | 0.043 |
| ENSG00000198265.11 | HELZ | 7.939 | 9.829 | 6.994 | -0.438 | 0.13 | -3.294 | 9.9e-4 | 0.044 |
| ENSG00000105404.10 | RABAC1 | 78.968 | 50.716 | 93.094 | 0.863 | 0.267 | 3.293 | 9.9e-4 | 0.044 |
| ENSG00000121741.16 | ZMYM2 | 23.26 | 28.638 | 20.571 | -0.458 | 0.134 | -3.29 | 0.001 | 0.044 |
| ENSG00000157020.17 | SEC13 | 95.61 | 77.252 | 104.789 | 0.435 | 0.155 | 3.289 | 0.001 | 0.044 |
| ENSG00000161642.17 | ZNF385A | 280.691 | 229.426 | 306.324 | 0.415 | 0.135 | 3.288 | 0.001 | 0.044 |
| ENSG00000270188.1 | MTRNR2L11 | 1.194 | 0.751 | 1.415 | 0.464 | 0.29 | 3.288 | 0.001 | 0.044 |
| ENSG00000140416.20 | TPM1 | 14.454 | 19.075 | 12.144 | -0.611 | 0.187 | -3.288 | 0.001 | 0.044 |
| ENSG00000130513.6 | GDF15 | 70.568 | 42.243 | 84.73 | 0.987 | 0.371 | 3.287 | 0.001 | 0.044 |
| ENSG00000163870.14 | TPRA1 | 24.506 | 20.121 | 26.699 | 0.391 | 0.144 | 3.287 | 0.001 | 0.044 |
| ENSG00000239264.8 | TXNDC5 | 42.574 | 30.63 | 48.545 | 0.647 | 0.23 | 3.286 | 0.001 | 0.044 |
| ENSG00000134256.12 | CD101 | 2.748 | 4.413 | 1.915 | -0.893 | 0.449 | -3.285 | 0.001 | 0.044 |
| ENSG00000058091.16 | CDK14 | 7.403 | 4.778 | 8.715 | 0.75 | 0.268 | 3.285 | 0.001 | 0.044 |
| ENSG00000114107.8 | CEP70 | 4.236 | 3.216 | 4.746 | 0.447 | 0.215 | 3.283 | 0.001 | 0.044 |
| ENSG00000149212.11 | SESN3 | 1.901 | 3.158 | 1.273 | -0.871 | 0.469 | -3.283 | 0.001 | 0.044 |
| ENSG00000138944.7 | SHISAL1 | 0.063 | 0.016 | 0.086 | 0.096 | 0.745 | 3.282 | 0.001 | 0.044 |
| ENSG00000095015.5 | MAP3K1 | 3.153 | 4.245 | 2.607 | -0.54 | 0.223 | -3.282 | 0.001 | 0.044 |
| ENSG00000162892.15 | IL24 | 2.265 | 1.187 | 2.803 | 0.798 | 0.379 | 3.281 | 0.001 | 0.044 |
| ENSG00000232433.2 | GXYLT1P3 | 1.398 | 3.525 | 0.335 | -1.761 | 0.997 | -3.279 | 0.001 | 0.044 |
| ENSG00000173641.17 | HSPB7 | 0.92 | 0.427 | 1.166 | 0.603 | 0.658 | 3.279 | 0.001 | 0.044 |
| ENSG00000140464.19 | PML | 25.438 | 31.078 | 22.617 | -0.442 | 0.096 | -3.278 | 0.001 | 0.044 |
| ENSG00000198162.12 | MAN1A2 | 9.779 | 11.628 | 8.855 | -0.358 | 0.101 | -3.278 | 0.001 | 0.044 |
| ENSG00000163082.9 | SGPP2 | 0.863 | 0.43 | 1.08 | 0.541 | 0.408 | 3.278 | 0.001 | 0.044 |
| ENSG00000225553.7 | PHF1 | 20.267 | 15.908 | 22.447 | 0.472 | 0.152 | 3.277 | 0.001 | 0.044 |
| ENSG00000163629.12 | PTPN13 | 1 | 1.479 | 0.76 | -0.494 | 0.453 | -3.274 | 0.001 | 0.045 |
| ENSG00000147324.10 | MFHAS1 | 56.503 | 46.516 | 61.497 | 0.395 | 0.14 | 3.271 | 0.001 | 0.045 |
| ENSG00000179144.4 | GIMAP7 | 1.87 | 3.34 | 1.134 | -1.024 | 0.458 | -3.271 | 0.001 | 0.045 |
| ENSG00000166750.9 | SLFN5 | 8.31 | 5.791 | 9.569 | 0.638 | 0.228 | 3.27 | 0.001 | 0.045 |
| ENSG00000167657.13 | DAPK3 | 21.313 | 17.07 | 23.435 | 0.435 | 0.165 | 3.268 | 0.001 | 0.046 |
| ENSG00000155849.15 | ELMO1 | 22.221 | 30.226 | 18.219 | -0.7 | 0.219 | -3.265 | 0.001 | 0.046 |
| ENSG00000213443.2 | AC007068.1 | 5.393 | 2.783 | 6.699 | 1.025 | 0.395 | 3.265 | 0.001 | 0.046 |
| ENSG00000265808.3 | SEC22B | 21.953 | 18.804 | 23.528 | 0.309 | 0.113 | 3.265 | 0.001 | 0.046 |
| ENSG00000079950.13 | STX7 | 91.887 | 79.712 | 97.974 | 0.294 | 0.113 | 3.263 | 0.001 | 0.046 |
| ENSG00000102595.19 | UGGT2 | 12.262 | 12.017 | 12.384 | 0.04 | 0.11 | 3.263 | 0.001 | 0.046 |
| ENSG00000121691.4 | CAT | 68.532 | 87.796 | 58.9 | -0.568 | 0.157 | -3.262 | 0.001 | 0.046 |
| ENSG00000071189.21 | SNX13 | 19.374 | 24.549 | 16.787 | -0.522 | 0.095 | -3.261 | 0.001 | 0.046 |
| ENSG00000123405.13 | NFE2 | 0.107 | 0.279 | 0.021 | -0.326 | 1.233 | -3.26 | 0.001 | 0.046 |
| ENSG00000172366.19 | MCRIP2 | 76.857 | 66.169 | 82.201 | 0.309 | 0.124 | 3.258 | 0.001 | 0.046 |
| ENSG00000161381.13 | PLXDC1 | 10.85 | 17.864 | 7.343 | -1.177 | 0.469 | -3.258 | 0.001 | 0.046 |
| ENSG00000165424.6 | ZCCHC24 | 1.962 | 2.731 | 1.578 | -0.533 | 0.279 | -3.258 | 0.001 | 0.046 |
| ENSG00000178386.12 | ZNF223 | 4.708 | 5.961 | 4.081 | -0.454 | 0.13 | -3.255 | 0.001 | 0.047 |
| ENSG00000105717.13 | PBX4 | 1.315 | 0.741 | 1.603 | 0.58 | 0.387 | 3.252 | 0.001 | 0.047 |
| ENSG00000179163.11 | FUCA1 | 87.425 | 131.491 | 65.392 | -0.997 | 0.288 | -3.249 | 0.001 | 0.048 |
| ENSG00000177951.17 | BET1L | 26.408 | 21.969 | 28.628 | 0.367 | 0.106 | 3.247 | 0.001 | 0.048 |
| ENSG00000074696.12 | HACD3 | 22.51 | 26.271 | 20.629 | -0.334 | 0.092 | -3.245 | 0.001 | 0.048 |
| ENSG00000143507.17 | DUSP10 | 9.023 | 6.851 | 10.109 | 0.501 | 0.187 | 3.243 | 0.001 | 0.048 |
| ENSG00000130283.8 | GDF1 | 0.111 | 0.252 | 0.041 | -0.267 | 0.83 | -3.243 | 0.001 | 0.048 |
| ENSG00000132623.15 | ANKEF1 | 24.167 | 44.241 | 14.13 | -1.58 | 0.455 | -3.24 | 0.001 | 0.049 |
| ENSG00000140545.14 | MFGE8 | 5.107 | 7.715 | 3.803 | -0.859 | 0.353 | -3.239 | 0.001 | 0.049 |
| ENSG00000172667.10 | ZMAT3 | 8.769 | 10.606 | 7.85 | -0.391 | 0.13 | -3.239 | 0.001 | 0.049 |
| ENSG00000111196.9 | MAGOHB | 11.498 | 8.64 | 12.927 | 0.531 | 0.154 | 3.238 | 0.001 | 0.049 |
| ENSG00000166471.10 | TMEM41B | 14.981 | 12.451 | 16.246 | 0.359 | 0.118 | 3.238 | 0.001 | 0.049 |
| ENSG00000151883.17 | PARP8 | 34.009 | 43.309 | 29.359 | -0.545 | 0.208 | -3.235 | 0.001 | 0.049 |
| ENSG00000227835.8 | CARM1P1 | 3.007 | 1.236 | 3.893 | 1.13 | 0.51 | 3.235 | 0.001 | 0.049 |
| ENSG00000160570.13 | DEDD2 | 24.438 | 17.347 | 27.984 | 0.66 | 0.205 | 3.233 | 0.001 | 0.049 |
| ENSG00000173846.12 | PLK3 | 55.088 | 40.041 | 62.612 | 0.632 | 0.204 | 3.233 | 0.001 | 0.049 |
| ENSG00000087074.7 | PPP1R15A | 72.586 | 59.74 | 79.009 | 0.397 | 0.134 | 3.232 | 0.001 | 0.049 |
| ENSG00000165695.9 | AK8 | 21.857 | 8.944 | 28.313 | 1.56 | 0.519 | 3.23 | 0.001 | 0.049 |

**Table S3. 69 biological process terms which significantly upregulated in moyamoya disease**

| **GO Term** | **Count** | **%** | ***P*Value** |
| --- | --- | --- | --- |
| apoptotic process | 23 | 7.232704402515723 | 1.5583609129609635E-4 |
| cellular response to glucose starvation | 7 | 2.20125786163522 | 1.6187211018344516E-4 |
| intrinsic apoptotic signaling pathway in response to endoplasmic reticulum stress | 6 | 1.8867924528301887 | 1.7323774656253206E-4 |
| response to unfolded protein | 7 | 2.20125786163522 | 1.7940608870419594E-4 |
| response to endoplasmic reticulum stress | 8 | 2.515723270440252 | 2.868336281115387E-4 |
| positive regulation of cell adhesion | 7 | 2.20125786163522 | 2.6507537778330137E-4 |
| negative regulation of cell proliferation | 18 | 5.660377358490567 | 6.833621049824309E-4 |
| ER to Golgi vesicle-mediated transport | 9 | 2.8301886792452833 | 8.357889758681169E-4 |
| endoplasmic reticulum unfolded protein response | 6 | 1.8867924528301887 | 0.0011244276595211776 |
| signal transduction | 34 | 10.69182389937107 | 0.0017068836604265275 |
| positive regulation of cell migration | 12 | 3.7735849056603774 | 0.001903830455079739 |
| response to ischemia | 6 | 1.8867924528301887 | 0.002846868184538378 |
| vesicle-mediated transport | 11 | 3.459119496855346 | 0.003045978591927707 |
| regulation of cell cycle | 13 | 4.088050314465408 | 0.0033546300372450746 |
| positive regulation of JNK cascade | 7 | 2.20125786163522 | 0.0037908324432386746 |
| glutamine metabolic process | 4 | 1.257861635220126 | 0.004292966618751579 |
| cellular response to hypoxia | 8 | 2.515723270440252 | 0.005561056717979312 |
| positive regulation of neutrophil chemotaxis | 4 | 1.257861635220126 | 0.006200580468190524 |
| negative regulation of apoptotic process | 17 | 5.345911949685535 | 0.006756100624089953 |
| retrograde vesicle-mediated transport, Golgi to ER | 5 | 1.5723270440251573 | 0.007399494751574143 |
| UDP-N-acetylglucosamine biosynthetic process | 3 | 0.9433962264150944 | 0.007689581407255733 |
| positive regulation of T cell mediated cytotoxicity | 4 | 1.257861635220126 | 0.007711893006006252 |
| brain development | 11 | 3.459119496855346 | 0.008456452225100537 |
| response to activity | 5 | 1.5723270440251573 | 0.010255114680828046 |
| neurogenesis | 5 | 1.5723270440251573 | 0.010255114680828046 |
| chemotaxis | 7 | 2.20125786163522 | 0.011206419409931945 |
| positive regulation of phosphorylation | 4 | 1.257861635220126 | 0.011336305306388425 |
| protein folding in endoplasmic reticulum | 3 | 0.9433962264150944 | 0.0115153974606595 |
| neuron development | 5 | 1.5723270440251573 | 0.011571553838156228 |
| positive regulation of filopodium assembly | 4 | 1.257861635220126 | 0.012371140183271945 |
| cellular response to cytokine stimulus | 4 | 1.257861635220126 | 0.012371140183271945 |
| response to calcium ion | 5 | 1.5723270440251573 | 0.014518872675138557 |
| angiogenesis | 10 | 3.1446540880503147 | 0.015064238652154921 |
| amino acid transmembrane transport | 4 | 1.257861635220126 | 0.0170376451822032 |
| response to axon injury | 4 | 1.257861635220126 | 0.0170376451822032 |
| negative regulation of epidermal growth factor-activated receptor activity | 3 | 0.9433962264150944 | 0.0184915390679002 |
| folic acid metabolic process | 3 | 0.9433962264150944 | 0.021125346923711 |
| negative regulation of epithelial cell apoptotic process | 3 | 0.9433962264150944 | 0.021125346923711 |
| protein phosphorylation | 15 | 4.716981132075472 | 0.021563779209397158 |
| cell cycle | 12 | 3.7735849056603774 | 0.021618615212824585 |
| inflammatory response | 13 | 4.088050314465408 | 0.02372106998894055 |
| amino acid transport | 4 | 1.257861635220126 | 0.0240732637490376 |
| activation of MAPK activity | 5 | 1.5723270440251573 | 0.02492015055831976 |
| iron ion homeostasis | 4 | 1.257861635220126 | 0.0256415738939427 |
| protein kinase C signaling | 3 | 0.9433962264150944 | 0.026824763987133286 |
| N-acetylglucosamine metabolic process | 3 | 0.9433962264150944 | 0.026824763987133286 |
| release of sequestered calcium ion into cytosol | 4 | 1.257861635220126 | 0.027263530955943725 |
| intracellular protein transport | 11 | 3.459119496855346 | 0.028486953253913944 |
| ovarian follicle development | 4 | 1.257861635220126 | 0.028939020447717432 |
| neutral amino acid transport | 3 | 0.9433962264150944 | 0.02988051112968474 |
| regulation of apoptotic process | 9 | 2.8301886792452833 | 0.030442413221904722 |
| response to lipopolysaccharide | 7 | 2.20125786163522 | 0.03186971281108597 |
| extracellular matrix disassembly | 4 | 1.257861635220126 | 0.03244995648266838 |
| response to osmotic stress | 3 | 0.9433962264150944 | 0.03306723514663719 |
| positive regulation of insulin secretion | 4 | 1.257861635220126 | 0.03428499635990627 |
| positive regulation of cytosolic calcium ion concentration | 7 | 2.20125786163522 | 0.03450157677070652 |
| response to hyperoxia | 3 | 0.9433962264150944 | 0.03638029025328055 |
| apoptotic cell clearance | 3 | 0.9433962264150944 | 0.03638029025328055 |
| positive regulation of angiogenesis | 7 | 2.20125786163522 | 0.039177216857523185 |
| vesicle docking | 3 | 0.9433962264150944 | 0.03981514059009477 |
| response to cytokine | 4 | 1.257861635220126 | 0.04214939772231394 |
| carbohydrate derivative metabolic process | 2 | 0.628930817610063 | 0.04480824244885797 |
| cell surface receptor signaling pathway involved in cell-cell signaling | 2 | 0.628930817610063 | 0.04480824244885797 |
| valine biosynthetic process | 2 | 0.628930817610063 | 0.04480824244885797 |
| negative regulation of adherens junction organization | 2 | 0.628930817610063 | 0.04480824244885797 |
| vascular associated smooth muscle cell migration | 2 | 0.628930817610063 | 0.04480824244885797 |
| positive regulation of protein kinase B signaling | 6 | 1.8867924528301887 | 0.04557578091673581 |
| cellular response to oxidative stress | 5 | 1.5723270440251573 | 0.048067025497390864 |
| negative regulation of autophagy | 4 | 1.257861635220126 | 0.048588641155803865 |

**Table S4. 35 biological process terms which significantly downregulated in moyamoya disease**

| **Term** | **Count** | **%** | **PValue** |
| --- | --- | --- | --- |
| defense response to virus | 15 | 5.952380952380952 | 8.186297055912488E-7 |
| response to virus | 11 | 4.365079365079365 | 9.090548715704141E-7 |
| negative regulation of viral genome replication | 6 | 2.380952380952381 | 2.1842433118414727E-4 |
| innate immune response | 18 | 7.142857142857142 | 9.085758931170474E-4 |
| cellular response to corticotropin-releasing hormone stimulus | 3 | 1.1904761904761905 | 0.0013991631838022197 |
| mast cell degranulation | 4 | 1.5873015873015872 | 0.0019319248587016927 |
| interleukin-27-mediated signaling pathway | 3 | 1.1904761904761905 | 0.0028919879717957727 |
| positive regulation of NF-kappaB transcription factor activity | 8 | 3.1746031746031744 | 0.0032329185028712286 |
| cell surface receptor signaling pathway | 11 | 4.365079365079365 | 0.0039019421750506036 |
| inflammatory response | 13 | 5.158730158730158 | 0.004170361624442513 |
| phosphatidylinositol phosphorylation | 5 | 1.984126984126984 | 0.004549644635910996 |
| cellular response to fibroblast growth factor stimulus | 4 | 1.5873015873015872 | 0.00713091735698965 |
| response to interferon-alpha | 3 | 1.1904761904761905 | 0.007338340372142616 |
| defense response | 5 | 1.984126984126984 | 0.015045443250692812 |
| apoptotic process | 15 | 5.952380952380952 | 0.01516066119863811 |
| immune response | 13 | 5.158730158730158 | 0.015960795630034285 |
| positive regulation of apoptotic process | 10 | 3.968253968253968 | 0.017800545241890706 |
| MAPK cascade | 6 | 2.380952380952381 | 0.01916036794672791 |
| negative regulation of cell migration | 7 | 2.7777777777777777 | 0.01929388550510907 |
| positive regulation of regulatory T cell differentiation | 3 | 1.1904761904761905 | 0.019320059677388677 |
| cellular response to virus | 5 | 1.984126984126984 | 0.019939761041027144 |
| cellular response to exogenous dsRNA | 3 | 1.1904761904761905 | 0.021424461359911672 |
| cytoskeleton organization | 6 | 2.380952380952381 | 0.029172901968819633 |
| negative regulation of viral entry into host cell | 3 | 1.1904761904761905 | 0.030722442758426956 |
| myeloid cell differentiation | 3 | 1.1904761904761905 | 0.030722442758426956 |
| muscle organ development | 5 | 1.984126984126984 | 0.03239463833383423 |
| angiogenesis | 8 | 3.1746031746031744 | 0.03275348591567115 |
| regulation of response to reactive oxygen species | 2 | 0.7936507936507936 | 0.03556060282436133 |
| Rac protein signal transduction | 3 | 1.1904761904761905 | 0.03586640730846599 |
| response to interferon-gamma | 3 | 1.1904761904761905 | 0.03855412703596253 |
| positive regulation of tumor necrosis factor production | 5 | 1.984126984126984 | 0.041165093263470884 |
| negative regulation of interleukin-2 production | 3 | 1.1904761904761905 | 0.044150081461622026 |
| hydrogen peroxide catabolic process | 3 | 1.1904761904761905 | 0.044150081461622026 |
| protein localization to plasma membrane | 6 | 2.380952380952381 | 0.04485875452544011 |
| negative regulation of complement activation | 2 | 0.7936507936507936 | 0.04713207224436637 |

**Table S5. Summary of differential expression analysis using M2-induced iMG cells between progressive group and stable group in moyamoya disease**

| **ID** | **Symbol** | **Expression Mean** | **Expression progressive group** | **Expression stable group** | **log2FC** | **lfcSE** | **Stat** | ***p*-value** | **padj** |
| --- | --- | --- | --- | --- | --- | --- | --- | --- | --- |
| ENSG00000122641.10 | INHBA | 9.78 | 16.855 | 2.706 | -2.268 | 0.295 | -8.491 | 2.1e-17 | 4.7e-13 |
| ENSG00000241394.7 | HLA-DMA | 28.858 | 0 | 57.715 | 5.876 | 3.384 | 7.472 | 7.9e-14 | 9.1e-10 |
| ENSG00000206286.11 | VPS52 | 2.331 | 4.663 | 0 | -2.502 | 3.384 | -7.26 | 3.9e-13 | 3.0e-9 |
| ENSG00000276618.4 | RAD17 | 3.572 | 5.35 | 1.793 | -1.185 | 0.467 | -6.374 | 1.8e-10 | 1.1e-6 |
| ENSG00000144136.10 | SLC20A1 | 119.089 | 159.339 | 78.839 | -1.006 | 0.144 | -6.274 | 3.5e-10 | 1.6e-6 |
| ENSG00000244682.7 | FCGR2C | 29.807 | 48.682 | 10.931 | -2.058 | 0.332 | -6.175 | 6.6e-10 | 2.5e-6 |
| ENSG00000258947.6 | TUBB3 | 22.852 | 14.631 | 31.074 | 1.037 | 0.224 | 5.815 | 6.0e-9 | 2.0e-5 |
| ENSG00000170458.13 | CD14 | 26.791 | 45.494 | 8.087 | -2.355 | 0.407 | -5.587 | 2.3e-8 | 6.6e-5 |
| ENSG00000100453.12 | GZMB | 7.656 | 14.348 | 0.963 | -2.967 | 0.684 | -5.413 | 6.2e-8 | 1.6e-4 |
| ENSG00000196611.4 | MMP1 | 2.554 | 4.161 | 0.948 | -1.406 | 0.384 | -5.3 | 1.2e-7 | 2.7e-4 |
| ENSG00000170054.14 | SERPINA9 | 2.086 | 4.021 | 0.151 | -2.125 | 0.911 | -5.202 | 2.0e-7 | 4.1e-4 |
| ENSG00000173641.17 | HSPB7 | 1.166 | 1.998 | 0.335 | -1.168 | 0.443 | -5.176 | 2.3e-7 | 4.3e-4 |
| ENSG00000198286.9 | CARD11 | 0.908 | 1.279 | 0.536 | -0.569 | 0.449 | -4.993 | 5.9e-7 | 0.001 |
| ENSG00000087589.16 | CASS4 | 21.956 | 30.604 | 13.308 | -1.143 | 0.219 | -4.95 | 7.4e-7 | 0.001 |
| ENSG00000086289.11 | EPDR1 | 33.44 | 23.501 | 43.378 | 0.857 | 0.196 | 4.943 | 7.7e-7 | 0.001 |
| ENSG00000100985.7 | MMP9 | 4855.943 | 3366.393 | 6345.493 | 0.914 | 0.208 | 4.868 | 1.1e-6 | 0.002 |
| ENSG00000139572.3 | GPR84 | 5.271 | 7.649 | 2.893 | -1.152 | 0.256 | -4.861 | 1.2e-6 | 0.002 |
| ENSG00000227222.7 | DHX16 | 1.19 | 1.858 | 0.522 | -0.908 | 0.617 | -4.824 | 1.4e-6 | 0.002 |
| ENSG00000123610.4 | TNFAIP6 | 13.382 | 21.291 | 5.473 | -1.784 | 0.368 | -4.807 | 1.5e-6 | 0.002 |
| ENSG00000162723.9 | SLAMF9 | 9.226 | 5.677 | 12.774 | 1.045 | 0.276 | 4.742 | 2.1e-6 | 0.002 |
| ENSG00000123689.5 | G0S2 | 88.092 | 137.522 | 38.663 | -1.804 | 0.363 | -4.733 | 2.2e-6 | 0.002 |
| ENSG00000100100.12 | PIK3IP1 | 15.159 | 11.991 | 18.327 | 0.573 | 0.144 | 4.732 | 2.2e-6 | 0.002 |
| ENSG00000152767.16 | FARP1 | 52.786 | 34.941 | 70.63 | 0.995 | 0.217 | 4.702 | 2.6e-6 | 0.003 |
| ENSG00000278555.4 | LILRB4 | 54.792 | 0.272 | 109.311 | 6.438 | 1.81 | 4.644 | 3.4e-6 | 0.003 |
| ENSG00000283976.1 | AC244216.3 | 1.078 | 0.003 | 2.153 | 1.652 | 1.596 | 4.597 | 4.3e-6 | 0.004 |
| ENSG00000136603.13 | SKIL | 5.74 | 7.441 | 4.038 | -0.744 | 0.204 | -4.585 | 4.5e-6 | 0.004 |
| ENSG00000277594.2 | DDX52 | 3.079 | 5.401 | 0.758 | -1.864 | 0.488 | -4.535 | 5.8e-6 | 0.005 |
| ENSG00000140961.12 | OSGIN1 | 21.341 | 15.62 | 27.061 | 0.756 | 0.179 | 4.532 | 5.8e-6 | 0.005 |
| ENSG00000278243.2 | TSTA3 | 4.565 | 7.387 | 1.743 | -1.612 | 0.277 | -4.52 | 6.2e-6 | 0.005 |
| ENSG00000130203.9 | APOE | 2563.896 | 1900.553 | 3227.24 | 0.764 | 0.201 | 4.508 | 6.5e-6 | 0.005 |
| ENSG00000113070.7 | HBEGF | 22.488 | 36.689 | 8.288 | -2.021 | 0.455 | -4.481 | 7.4e-6 | 0.005 |
| ENSG00000126947.12 | ARMCX1 | 11.38 | 9.142 | 13.618 | 0.527 | 0.149 | 4.471 | 7.8e-6 | 0.006 |
| ENSG00000196549.10 | MME | 4.98 | 7.735 | 2.224 | -1.438 | 0.305 | -4.418 | 1.0e-5 | 0.007 |
| ENSG00000117525.13 | F3 | 107.991 | 145.916 | 70.065 | -1.048 | 0.22 | -4.415 | 1.0e-5 | 0.007 |
| ENSG00000274669.5 | LILRB1 | 6.779 | 11.676 | 1.881 | -2.137 | 0.419 | -4.395 | 1.1e-5 | 0.007 |
| ENSG00000111802.13 | TDP2 | 97.098 | 85.112 | 109.083 | 0.354 | 0.107 | 4.389 | 1.1e-5 | 0.007 |
| ENSG00000152413.14 | HOMER1 | 8.126 | 9.466 | 6.786 | -0.427 | 0.161 | -4.375 | 1.2e-5 | 0.008 |
| ENSG00000117984.13 | CTSD | 5735.253 | 4298.948 | 7171.558 | 0.738 | 0.187 | 4.351 | 1.4e-5 | 0.008 |
| ENSG00000129925.10 | TMEM8A | 61.457 | 53.01 | 69.905 | 0.393 | 0.116 | 4.321 | 1.6e-5 | 0.009 |
| ENSG00000152229.18 | PSTPIP2 | 5.694 | 7.561 | 3.827 | -0.827 | 0.261 | -4.291 | 1.8e-5 | 0.01 |
| ENSG00000274129.4 | TSEN34 | 2.234 | 0.166 | 4.302 | 2.185 | 0.955 | 4.241 | 2.2e-5 | 0.012 |
| ENSG00000281965.1 | AC243791.2 | 1.966 | 1.025 | 2.907 | 0.948 | 0.37 | 4.221 | 2.4e-5 | 0.013 |
| ENSG00000203485.12 | INF2 | 80.825 | 72.584 | 89.067 | 0.292 | 0.112 | 4.216 | 2.5e-5 | 0.013 |
| ENSG00000133048.12 | CHI3L1 | 3515.585 | 2503.745 | 4527.425 | 0.854 | 0.233 | 4.215 | 2.5e-5 | 0.013 |
| ENSG00000162522.10 | KIAA1522 | 12.124 | 14.723 | 9.524 | -0.579 | 0.136 | -4.183 | 2.9e-5 | 0.015 |
| ENSG00000115457.9 | IGFBP2 | 8.105 | 14.239 | 1.971 | -2.359 | 0.586 | -4.173 | 3.0e-5 | 0.015 |
| ENSG00000166557.12 | TMED3 | 78.703 | 69.775 | 87.63 | 0.325 | 0.103 | 4.167 | 3.1e-5 | 0.015 |
| ENSG00000225217.1 | HSPA7 | 9.833 | 15.84 | 3.827 | -1.803 | 0.467 | -4.141 | 3.5e-5 | 0.017 |
| ENSG00000163528.12 | CHCHD4 | 11.27 | 14.245 | 8.295 | -0.714 | 0.165 | -4.135 | 3.5e-5 | 0.017 |
| ENSG00000227500.9 | SCAMP4 | 44.517 | 39.307 | 49.728 | 0.332 | 0.102 | 4.111 | 3.9e-5 | 0.018 |
| ENSG00000276146.4 | LILRB2 | 5.74 | 10.851 | 0.629 | -2.863 | 0.943 | -4.108 | 4.0e-5 | 0.018 |
| ENSG00000121316.10 | PLBD1 | 113.687 | 92.924 | 134.451 | 0.528 | 0.138 | 4.09 | 4.3e-5 | 0.019 |
| ENSG00000173110.7 | HSPA6 | 4.049 | 5.986 | 2.112 | -1.167 | 0.341 | -4.077 | 4.6e-5 | 0.02 |
| ENSG00000258984.5 | UBE2F-SCLY | 0.529 | 0.145 | 0.913 | 0.74 | 1.046 | 4.051 | 5.1e-5 | 0.022 |
| ENSG00000182809.10 | CRIP2 | 1.098 | 1.911 | 0.285 | -1.18 | 0.623 | -4.04 | 5.3e-5 | 0.022 |
| ENSG00000285259.1 | AC270285.8 | 11056.24 | 8964.505 | 13147.975 | 0.552 | 0.161 | 4.035 | 5.5e-5 | 0.022 |
| ENSG00000106330.11 | MOSPD3 | 9.846 | 7.996 | 11.696 | 0.497 | 0.157 | 4.007 | 6.1e-5 | 0.025 |
| ENSG00000179588.8 | ZFPM1 | 1.199 | 0.963 | 1.435 | 0.311 | 0.243 | 3.999 | 6.4e-5 | 0.025 |
| ENSG00000139567.12 | ACVRL1 | 4.268 | 5.632 | 2.904 | -0.765 | 0.349 | -3.996 | 6.4e-5 | 0.025 |
| ENSG00000182853.11 | VMO1 | 5.039 | 7.681 | 2.398 | -1.353 | 0.399 | -3.985 | 6.8e-5 | 0.025 |
| ENSG00000101665.8 | SMAD7 | 11.039 | 15.002 | 7.076 | -0.986 | 0.167 | -3.983 | 6.8e-5 | 0.025 |
| ENSG00000014257.15 | ACPP | 1.581 | 2.277 | 0.885 | -0.798 | 0.378 | -3.98 | 6.9e-5 | 0.025 |
| ENSG00000110719.9 | TCIRG1 | 823.25 | 717.649 | 928.85 | 0.372 | 0.116 | 3.977 | 7.0e-5 | 0.025 |
| ENSG00000115993.12 | TRAK2 | 21.361 | 19.432 | 23.29 | 0.25 | 0.093 | 3.976 | 7.0e-5 | 0.025 |
| ENSG00000116016.13 | EPAS1 | 105.471 | 95.895 | 115.048 | 0.26 | 0.095 | 3.966 | 7.3e-5 | 0.026 |
| ENSG00000075340.22 | ADD2 | 3.164 | 1.779 | 4.55 | 0.998 | 0.353 | 3.945 | 8.0e-5 | 0.028 |
| ENSG00000120756.12 | PLS1 | 1.012 | 1.468 | 0.556 | -0.665 | 0.547 | -3.935 | 8.3e-5 | 0.028 |
| ENSG00000140526.17 | ABHD2 | 116.28 | 151.819 | 80.74 | -0.903 | 0.216 | -3.92 | 8.9e-5 | 0.03 |
| ENSG00000196189.12 | SEMA4A | 7.654 | 10.404 | 4.905 | -0.95 | 0.272 | -3.913 | 9.1e-5 | 0.03 |
| ENSG00000213699.8 | SLC35F6 | 19.745 | 16.318 | 23.172 | 0.481 | 0.168 | 3.867 | 1.1e-4 | 0.036 |
| ENSG00000103569.9 | AQP9 | 198.416 | 242.836 | 153.996 | -0.654 | 0.154 | -3.865 | 1.1e-4 | 0.036 |
| ENSG00000120162.9 | MOB3B | 8.709 | 10.691 | 6.726 | -0.598 | 0.153 | -3.855 | 1.2e-4 | 0.037 |
| ENSG00000108688.11 | CCL7 | 126.313 | 222.572 | 30.054 | -2.848 | 0.708 | -3.848 | 1.2e-4 | 0.037 |
| ENSG00000172927.7 | MYEOV | 11.444 | 16.892 | 5.997 | -1.355 | 0.515 | -3.833 | 1.3e-4 | 0.039 |
| ENSG00000082269.16 | FAM135A | 3.552 | 4.188 | 2.916 | -0.406 | 0.158 | -3.83 | 1.3e-4 | 0.039 |
| ENSG00000197879.15 | MYO1C | 76.642 | 69.564 | 83.72 | 0.264 | 0.117 | 3.811 | 1.4e-4 | 0.042 |
| ENSG00000171724.2 | VAT1L | 0.17 | 0.337 | 0.003 | -0.415 | 1.477 | -3.805 | 1.4e-4 | 0.042 |
| ENSG00000185291.11 | IL3RA | 5.088 | 7.557 | 2.619 | -1.242 | 0.43 | -3.805 | 1.4e-4 | 0.042 |
| ENSG00000278605.4 | TSEN34 | 5.407 | 0.033 | 10.781 | 3.511 | 1.966 | 3.783 | 1.6e-4 | 0.045 |
| ENSG00000277943.4 | CCL4 | 255.991 | 367.687 | 144.296 | -1.343 | 0.33 | -3.781 | 1.6e-4 | 0.045 |
| ENSG00000183019.7 | MCEMP1 | 0.731 | 1.284 | 0.179 | -0.954 | 0.819 | -3.769 | 1.6e-4 | 0.046 |
| ENSG00000157601.13 | MX1 | 9.723 | 12.647 | 6.799 | -0.807 | 0.462 | -3.76 | 1.7e-4 | 0.048 |

**Table S6. 29 biological process terms which significantly upregulated in the progressive group**

| **Term** | **Count** | **%** | ***P*Value** |
| --- | --- | --- | --- |
| positive regulation of cell migration | 7 | 12.5 | 5.681134042603261E-5 |
| cytokine-mediated signaling pathway | 6 | 10.714285714285714 | 5.691508156763721E-5 |
| signal transduction | 13 | 23.214285714285715 | 8.998631451259329E-5 |
| cell-cell signaling | 6 | 10.714285714285714 | 3.4952483275939956E-4 |
| activin receptor signaling pathway | 3 | 5.357142857142857 | 0.0013984925747130357 |
| inflammatory response | 6 | 10.714285714285714 | 0.004535836982999001 |
| positive regulation of pathway-restricted SMAD protein phosphorylation | 3 | 5.357142857142857 | 0.007768095668832268 |
| immune response-inhibiting cell surface receptor signaling pathway | 2 | 3.571428571428571 | 0.007924960408641867 |
| extrinsic apoptotic signaling pathway | 3 | 5.357142857142857 | 0.008380302454221545 |
| cell surface receptor signaling pathway | 5 | 8.928571428571429 | 0.00859734959709884 |
| negative regulation of BMP signaling pathway | 3 | 5.357142857142857 | 0.010686912715891833 |
| positive regulation of gene expression | 6 | 10.714285714285714 | 0.01128969846074324 |
| positive regulation of T cell proliferation | 3 | 5.357142857142857 | 0.012861374922930886 |
| positive regulation of interleukin-8 production | 3 | 5.357142857142857 | 0.012861374922930886 |
| Fc receptor mediated inhibitory signaling pathway | 2 | 3.571428571428571 | 0.01317402548777976 |
| cellular response to lipopolysaccharide | 4 | 7.142857142857142 | 0.01451099363218494 |
| positive regulation of natural killer cell chemotaxis | 2 | 3.571428571428571 | 0.015788337715262245 |
| G1/S transition of mitotic cell cycle | 3 | 5.357142857142857 | 0.016450925923149784 |
| positive regulation of interferon-gamma production | 3 | 5.357142857142857 | 0.018608811493434983 |
| defense response | 3 | 5.357142857142857 | 0.018608811493434983 |
| neutrophil chemotaxis | 3 | 5.357142857142857 | 0.019957867165563352 |
| cellular response to UV-A | 2 | 3.571428571428571 | 0.02875838760013415 |
| transforming growth factor beta receptor signaling pathway | 3 | 5.357142857142857 | 0.029400008052379096 |
| wound healing, spreading of epidermal cells | 2 | 3.571428571428571 | 0.03389935649617771 |
| response to virus | 3 | 5.357142857142857 | 0.034954567790008155 |
| negative regulation of calcium ion transport | 2 | 3.571428571428571 | 0.036459826944946844 |
| eosinophil chemotaxis | 2 | 3.571428571428571 | 0.04410138599297961 |
| negative regulation of cell growth | 3 | 5.357142857142857 | 0.04460776862453481 |
| positive regulation of protein kinase B signaling | 3 | 5.357142857142857 | 0.04524067644049085 |

**Table S7. 4 biological process terms which significantly downregulated in the progressive group**

| **Term** | **Count** | **%** | ***P*Value** |
| --- | --- | --- | --- |
| lipoprotein catabolic process | 2 | 6.451612903225806 | 0.007250098907122426 |
| hematopoietic stem cell homeostasis | 2 | 6.451612903225806 | 0.03574690062091243 |
| leukocyte migration | 2 | 6.451612903225806 | 0.042746893538515615 |
| antigen processing and presentation of exogenous peptide antigen via MHC class II | 2 | 6.451612903225806 | 0.042746893538515615 |
