# Supplementary material for: Angiogenic and inflammatory responses in human induced microglia-like (iMG) cells from patients with Moyamoya disease
Source: Sci Rep. 2023 Sep 8;13:14842. doi: 10.1038/s41598-023-41456-z (PMC10491754; doi:10.1038/s41598-023-41456-z)
Supplement: Supplementary file 3 — Supplementary Legends. [file 41598_2023_41456_MOESM3_ESM.docx]

Figure S1

Expression of the *RNF213* was measured by qPCR in iMG cells and monocyte. Fold changes were measured using the ΔΔ Ct method. The relative expression (fold change) of *RNF213* was not significantly different in resting, M1-, M2-induced iMG cells and monocyte in comparison to MMD and control group.

Figure S2

Expression of the angiogenic factors *MMP9*, *VEGFA*, and *TGFB1* was measured by qPCR in iMG cells and monocytes. Fold changes were measured using the ΔΔ Ct method.

The relative expression (fold change) of *MMP9* expression was significantly upregulated in resting iMG cells in MMD. The relative expression (fold change) of *VEGFA* was not significantly different in resting, M1-induced iMG cells and monocytes in comparison to MMD and control group. The relative expression (fold change) of *TGFB1* expression was significantly lower in monocytes of MMD.

Figure S3

Expression of the angiogenic factors *MMP9*, *VEGFA*, and *TGFB1* was measured by qPCR of resting, M1- and M2-induced iMG cells in the progressive and stable groups. Fold changes were measured using the ΔΔ Ct method.

The relative expression (fold change) of angiogenic factors *MMP9*, *VEGFA*, and *TGFB1* were not significantly different in resting, M1-induced iMG cells and monocytes in comparison to the progressive and stable groups.
